# The nutritional and metabolic impact of night shift work in a real-world setting

**DOI:** 10.1038/s44323-026-00107-y

**Published:** 2026-09-29

**Authors:** Friedrich C. Jassil, Nicholas E. Phillips, Alexandra Hemmer, Céline Joris, Andrew D. Biancolin, Steffen L. Hartmeyer, Victor Dorribo, Stephen Perrig, Laurence Genton, Virginie Sterpenich, Marcel Salathé, Jacques A. Pralong, Marilyne Andersen, Claude Pichard, Charna Dibner, Tinh-Hai Collet

**Affiliations:** 1 Service of Endocrinology, Diabetology, and Metabolism, Department of Medicine, Geneva University Hospitals, Geneva, Switzerland; 2 Diabetes Centre, Faculty of Medicine, University of Geneva, Geneva, Switzerland; 3 Centre for Obesity Research, University College London, London, UK; 4 Institute for Clinical Diabetology, German Diabetes Center, Leibniz Center for Diabetes Research at Heinrich-Heine-University Düsseldorf, Düsseldorf, Germany; 5 The Thoracic and Endocrine Surgery Division, Department of Surgery, Geneva University Hospitals, Geneva, Switzerland; 6 Department of Cell Physiology and Metabolism, Faculty of Medicine, University of Geneva, Geneva, Switzerland; 7 Laboratories of Neuroimmunology, Center for Research in Neuroscience and Service of Neurology, Department of Clinical Neurosciences, Lausanne University Hospital and University of Lausanne, Lausanne, Switzerland; 8 iGE3 Center, Geneva, Switzerland; 9 Laboratory of Integrated Performance in Design (LIPID), School of Architecture, Civil and Environmental Engineering (ENAC), EPFL (École Polytechnique Fédérale de Lausanne), Lausanne, Switzerland; 10 Department of Occupational and Environmental Health, Center for Primary Care and Public Health (Unisanté), University of Lausanne, Épalinges, Switzerland; 11 Service of Neurology, Geneva University Hospitals, Geneva, Switzerland; 12 Clinical Nutrition Unit, Service of Gastro-enterology, Hepatology and Nutrition, Geneva University Hospitals, Geneva, Switzerland; 13 Department of Neuroscience, Faculty of Medicine, University of Geneva, Geneva, Switzerland; 14 Digital Epidemiology Lab, School of Life Science, School of Computer and Communication Sciences, EPFL (École Polytechnique Fédérale de Lausanne), Lausanne, Switzerland; 15 Faculty of Medicine, University of Geneva, Geneva, Switzerland; 16 SwissMedPro Health Services, Geneva, Switzerland; 17 La Tour Hospital, Meyrin-Geneva, Switzerland

**Keywords:** Health care, Medical research, Physiology

## Abstract

Growing evidence from epidemiological and lab-controlled studies links night shift work to adverse health outcomes, but evidence from real-world occupational environments remains limited. Using multiple wearable devices and mobile health apps, alongside other objective and subjective assessment tools, we assess nutritional intake and cardiometabolic parameters among 72 shift workers recruited from a single-center Swiss hospital (82% female; median age 31 years; body mass index 22.7 kg/m^2^) during three consecutive night shifts and three consecutive day shifts. While intake of calories, protein, and fat is reduced during night shifts, glycemic variability and postprandial glucose spikes are increased compared to day shifts. After three consecutive night shifts, plasma cortisol is reduced, and the 24-h heart rate and heart rate variability are altered. Exploratory analysis reveals higher protein intake correlates with lower glycemic variability, whereas nighttime eating is associated with increased glycemic variability. Future interventional studies should explore whether adjusting meal composition and timing could improve glycemic variability in night shift workers.

## Introduction

According to the European Working Conditions Survey 2024, shift workers account for 21% of employees in the European Union^1^. In Switzerland, approximately 600,000 employees are subjected to irregular working hours, such as rotating shifts (a series of day shifts, evening shifts, and night shifts, then rotating from one type of shift to the next) or permanent night shifts^2^. These work patterns are common in sectors such as human health and social work services, transport and warehousing services, and accommodation and catering services^2^. Night shift work, associated with nocturnal light exposure, late meal consumption, and reduced sleep duration, can desynchronize endogenous body clocks from environmental cues, leading to circadian misalignment^3^. Epidemiological evidence indicates that prolonged exposure to night shift work increases susceptibility to obesity, type 2 diabetes, hypertension, and metabolic syndrome, leading to long-term complications such as cancer and cardiovascular mortality^4–6^.

Circadian misalignment protocols under lab-controlled conditions simulating shift work have provided important insights into the mechanisms underlying the negative consequences induced by night shift work^7–12^. A 3-day simulated night shift work led to decreased muscle insulin sensitivity and increased fasting plasma glucose levels^12^. Similarly, an 8-day simulated night shift resulted in elevated postprandial glucose, despite a concomitant increase in insulin levels^7^. Circadian misalignment also led to increased blood pressure (BP) and impaired attention, the latter being associated with increased sleepiness^9,11^. Interestingly, the circadian misalignment protocol in healthy chronic shift workers resulted in impaired glucose tolerance and reduced insulin sensitivity. Circadian misalignment also led to reduced total sleep time and increased 24-h BP^8,10^. However, it is important to note that the tightly controlled lab conditions do not fully replicate the various environmental and behavioral factors present in real-life settings of shift workers that can impact circadian rhythms. These factors include the variation in exposure to ambient light and temperature, the actual workload, physical and emotional exhaustion, variability in dietary and physical activity patterns, as well as smoking and caffeine intake^3,5^. Therefore, data from observational studies within real-world occupational settings are needed to bridge the knowledge gap between epidemiological and lab-controlled experimental evidence^5^.

Recent advances in wearable technology and mobile health applications (apps) have allowed for live data collection rather than retrospective self-reports, providing more accurate and real-time insights into health parameters and behaviors^13^. These technologies include continuous glucose monitors (CGM) to assess glycemic profiles, triaxial actigraphy devices to measure physical activity, sleep-wake cycles, and heart rate (HR), ambient light sensors to track light exposure, body temperature sensors, and smartphone food diary apps to record food intake. Together, these tools provide continuous 24-h insights into in vivo clock parameters during night shift work, addressing the limitations of earlier studies that relied on data collected from isolated time points^5^. A deeper understanding of the physiological changes linked to lifestyle behaviors during night shift work may steer the development of targeted occupational health interventions to improve the health and well-being of night shift workers.

Using multiple wearable devices and mobile health apps, alongside other objective and subjective assessment tools, we aimed to evaluate the nutritional and metabolic impacts of night shift work in a real-world environment in the OPTI-SHIFT observational study (Supplementary Fig. 1, ClinicalTrials.gov registry no. NCT05177965). The primary objective was to assess the differences in nutritional intake and glucose excursion between a series of day shifts and a series of night shifts. For secondary objectives, we compared cardiometabolic, physical activity, sleep quality, cognitive performance, mental health, and well-being between both shift periods. Other outcome data on light exposure and core body temperature from the same cohort have recently been published elsewhere^14^. We hypothesized that night shift exposure would adversely affect nutritional intake and glucose regulation, and contribute to unfavorable changes in cardiometabolic, behavioral, physiological, and psychological outcomes compared with day shifts.

## Results

### Participants’ characteristics and shift work patterns

We enrolled 72 night shift workers, consisting of 96% of healthcare workers. Of all participants, 82% were female with a median age of 31 (IQR 28 to 35) years and a body mass index (BMI) of 22.7 (IQR 20.4 to 26.2) kg/m^2^ (Table 1 and Supplementary Table 1). Two participants did not complete the series of day shifts due to withdrawal (*n* = 1) and loss of follow-up (*n* = 1). A participant flow diagram is shown in Supplementary Fig. 2. The self-reported shift schedules were between 21:40 h ± 01:12 h and 07:30 h ± 00:42 h for night shifts, and between 07:00 h ± 00:36 h and 16:30 h ± 01:42 h for day shifts. The median interval period between the series of night shifts (first time point) and the series of day shifts (second time point) was 10.3 (IQR 5.3 to 18.6) weeks. Participants reported an average of five years working in shift work, with three years in the current shift pattern averaging 40 h per week. The detailed work patterns, shift work systems, motivations, and workload are provided in Supplementary Table 2.

**Table 1:** Participant characteristics

Participant characteristics	*n* = 72
Age, years, median (IQR)	31 (28; 35)
Women, *n* (%)	59 (81.9)
Marital status, *n* (%)	
Single	37 (51.3)
Married, partnership, living with partner	31 (43.1)
Separated, divorced	4 (5.6)
*Employment and shift work*	
Profession, *n* (%)	
Nurse	36 (50.0)
Physician	20 (27.8)
Paramedic	13 (18.1)
Others	3 (4.1)
Duration of employment, years, median (IQR)	6 (3.75; 11)
Duration of shift work employment	5 (2.9; 10)
Duration of working in the present shift	3 (1.3; 5.5)
Regularity of shift work system, *n* (%)	
Regular	8 (11.1)
Irregular	57 (79.2)
Flexible	7 (9.7)
*Clinical data*	
Weight, kg, median (IQR)	64.4 (54.0; 75.1)
Height, m, median (IQR)	1.65 (1.60; 1.73)
Body mass index, kg/m^2^, median (IQR)	22.7 (20.4; 26.2)
Waist circumference, cm, median (IQR)	77.4 (71.4; 86.1)
Waist-to-hip ratio, median (IQR)	0.83 (0.78; 0.88)
Body composition by bioelectrical impedance analysis, median (IQR)	
Fat mass (kg)	17.5 (14.2; 24.4)
Fat-free mass (kg)	43.3 (39.3; 48.9)
Fat mass index (kg/m^2^)	6.2 (5.2; 8.3)
Fat-free mass index (kg/m^2^)	15.9 (14.8; 17.4)
Sleep quality^a^ (PSQI score, *n* = 69), mean ± SD	7.72 ± 3.57

^a^Sleep quality in the past month was assessed by the Pittsburgh Sleep Quality Index (PSQI), with a total score range of 1–21, higher scores indicating poorer sleep quality^72^. See also Supplementary Table 1.

### Night shifts reduced calorie, protein, and fat intake, with unchanged carbohydrate intake and altered 24-h timing of consumption

**Fig. 1:**
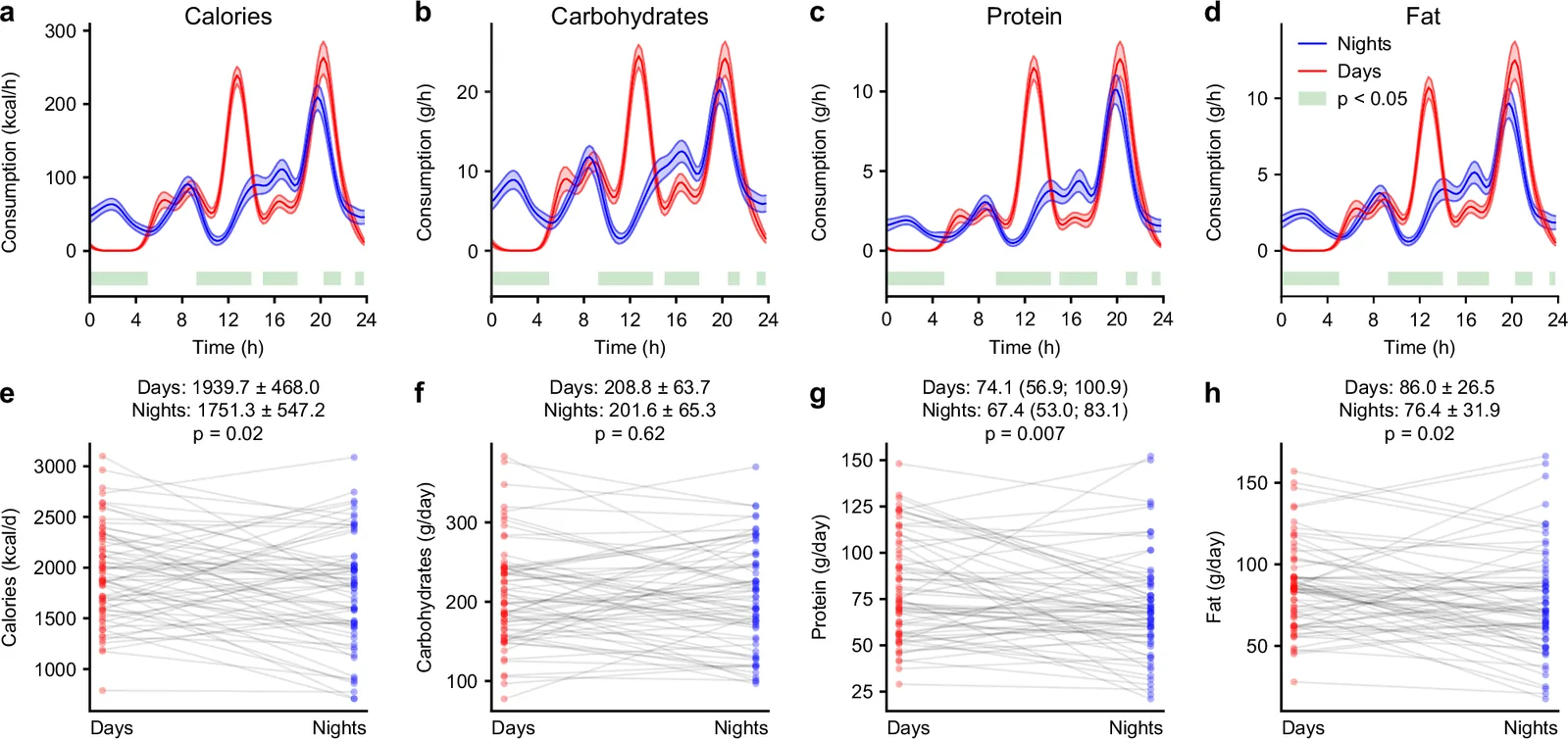
Night shifts were associated with lower calorie, protein, and fat intake, but similar carbohydrate intake compared to day shifts, with significant changes in the timing of consumption across 24 h. **a**–**d** The mean consumption rate per hour for night shifts (blue) and day shifts (red) of total calories, carbohydrates, protein, and fat, respectively. The shaded area represents the mean ± SEM, and the green bar represents significant group differences as assessed from *p* < 0.05 in a Wilcoxon non-parametric test, adjusted for multiple testing with the Benjamini-Hochberg procedure. **e**–**h** Individual average daily intake for the day (red) and night shift (blue) period of total calories, carbohydrates, protein, and fat, respectively. Group means ± SD are indicated above each panel, except for protein intake as median + IQR owing to non-normality. *n* = 66 for all analyses. See also Supplementary Table 3 and Supplementary Fig. 3.

Night shifts were associated with significant temporal changes in food and drink consumption according to the time of day, with higher nighttime calorie intake and reduced lunchtime intake (Fig. 1a). These trends were overall similar across calories, carbohydrates, protein, fat (Fig. 1a–d), and fiber (Supplementary Fig. 3). However, relative nighttime carbohydrate consumption was higher than protein and fat during night shifts as opposed to day shifts. Visualization of the hourly consumption rate comparing carbohydrates and protein (Fig. 1b, c) showed that relative nighttime carbohydrate intake was higher than protein intake during night shifts. Considering total consumption per day (i.e., the area under the curve of the consumption rates in Fig. 1 upper panels), night shifts were associated with slightly lower consumption of calories (mean −164 ± 530 kcal/day, *p* = 0.015, Fig. 1e), protein (mean −9.6 ± 28.1 g/day, *p* = 0.007, Fig. 1g) and fat (mean −8.9 ± 29.3 g/day, *p* = 0.017, Fig. 1h), and similar carbohydrate intake (mean −3.6 ± 59.2 g/day, *p* = 0.62, Fig. 1f).

This resulted in a higher proportion of carbohydrates during night shifts (+2.6%, IQR −1.3 to 8.6, *p* = 0.002, Supplementary Table 3). In comparison to the nutritional reference values for Switzerland^15^ (shown in Supplementary Table 3), the proportion of total calories from carbohydrate intake was slightly lower during day shifts. In both shift rotations, protein intake met the daily recommendation, whereas the proportion of fat intake exceeded the recommendation. While the intake of dietary fiber was slightly lower during night shift compared to day shift (−1.93 ± 6.36 g/day, *p* = 0.017), both did not meet the daily recommendation of 30 g.

These objective data corroborate the self-reported eating habits between day and night shifts (Supplementary Table 4). Lower subjective appetite (*p* = 0.008) and a reduced daily number of meals (*p* = 0.01), along with irregular mealtimes (*p* = 0.03), were reported during night shifts compared to day shifts. Dinner became the main meal of the day during night shifts (*p* = 0.004), with a higher frequency of eating in front of a screen during night shifts (*p* = 0.001). Lower consumption of alcohol was reported during night shifts compared to day shifts (*p* < 0.001), as confirmed with the smartphone food diary app (Supplementary Fig. 3).

### Night shifts are associated with lower 24-h mean glucose and increased nocturnal glycemic variability

**Fig. 2:**
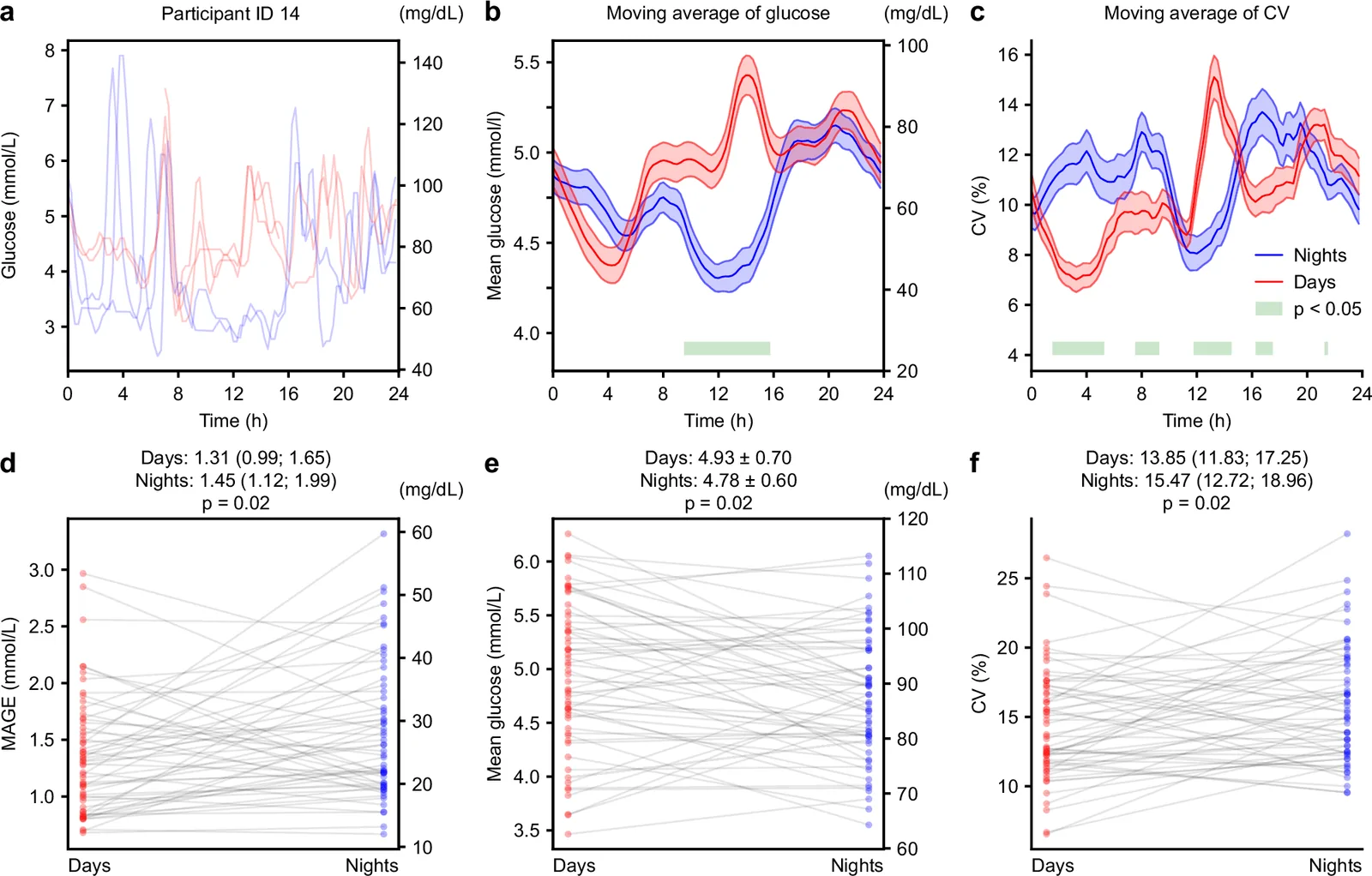
Night shift work is associated with significant differences in the glycemic mean and coefficient of variation (CV) across the time of day, with overall reduced glucose levels but higher glycemic variability. **a** Example of glucose levels as a function of the time of day for one participant (ID 14), where the different lines represent different study days. Blue, night shifts; red, day shifts. This selected example was chosen to illustrate the range of observations and does not represent the average participants’ response. **b**, **c** The 2-h moving average estimate over the 24-h clock of glucose mean and CV, respectively. The shaded area represents the mean ± SEM, and the green bar represents significant group differences as assessed from *p* < 0.05 in a Wilcoxon non-parametric test, adjusted for multiple testing with the Benjamini-Hochberg procedure. **d**–**f** Individual changes for mean amplitude of glycemic excursions (MAGE, **d**)^16^, and glucose mean (**e**) and CV (**f**). Group means ± SD are indicated above each panel, or group medians + IQR if not normally distributed. *n* = 58 for all analyses. See also Supplementary Table 5.

The CGM measures glucose as a function of the time of day across multiple study days (example shown in Fig. 2a), which allows us to estimate how the mean and short-term variation vary according to clock time, using e.g., a moving average. The moving average glucose levels comparing night and day shifts differed significantly as a function of the time of day, with the minimum night shift glucose levels at midday strongly diverging from the maximum glucose levels at 14:00 h during day shifts (Fig. 2b). The moving average glycemic variability differed significantly between shifts across multiple time blocks (green blocks, Fig. 2c), with a higher nocturnal coefficient of variation (CV) during night shifts but a lower CV in the early afternoon, possibly caused by lunch-skipping behavior during night shifts (Fig. 1a). Considering all data across the entire shift periods, night shifts were associated with a moderate decrease in the global mean glucose levels (difference −0.19 ± 0.61 mmol/L, *p* = 0.02, Fig. 2e) and a moderate increase in glycemic variability as assessed with both the global CV (difference +1.45% ± 4.5%, *p* = 0.02, Fig. 2f) and the mean amplitude of glycemic excursions (MAGE: difference +0.21 ± 0.64 mmol/L, *p* = 0.02, Fig. 2d)^16^.

### Night shift work is associated with lower baseline glucose levels but more pronounced postprandial glucose spikes

**Fig. 3:**
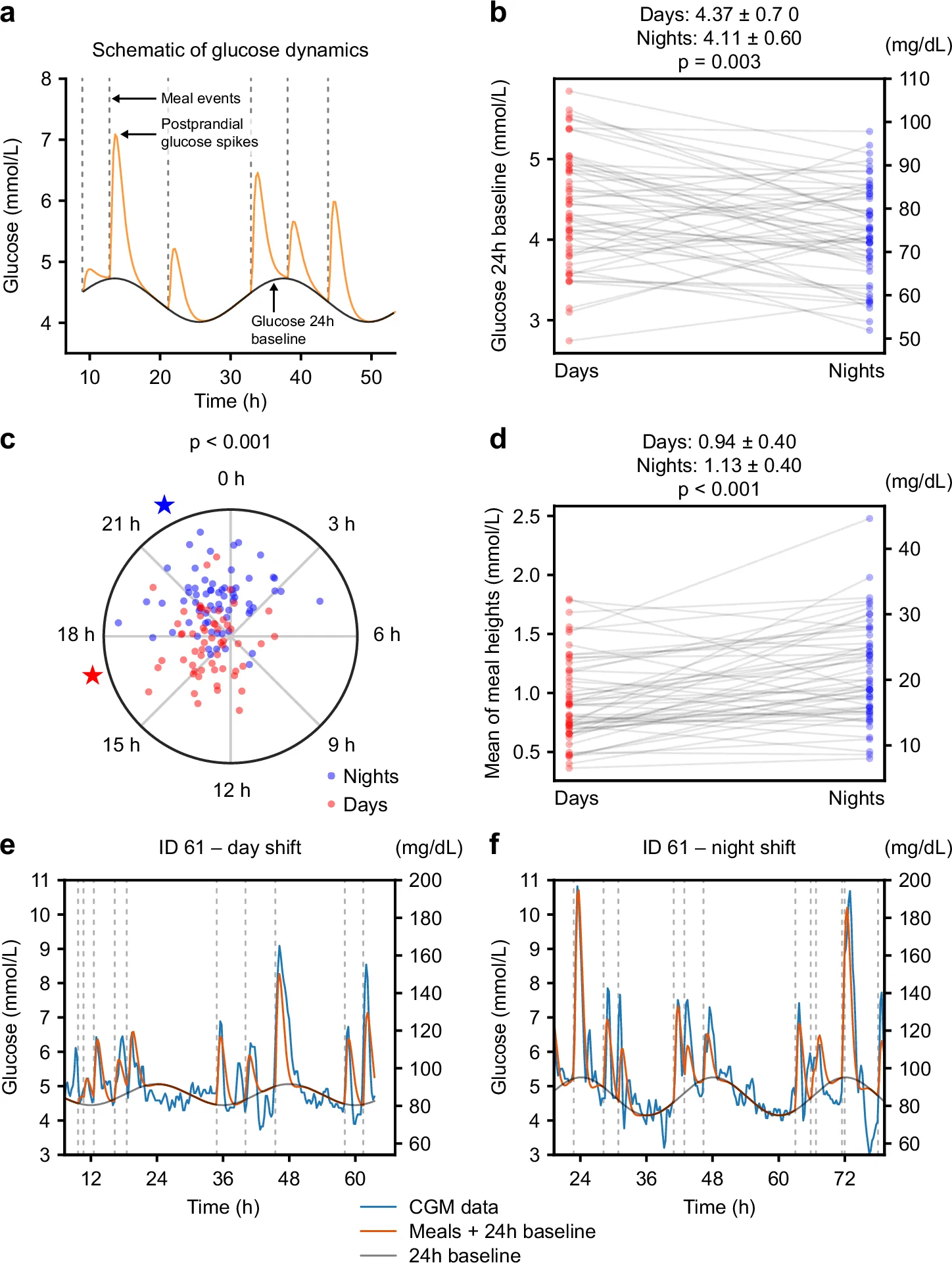
Dynamical modeling of glucose levels reveals that night shift work is associated with a lower baseline glucose level and higher average postprandial glucose spikes. **a** Schematic of the mathematical framework, which uses meal events (dashed lines) to model glucose levels as a superposition of postprandial glucose spikes (orange) on top of an underlying 24-h rhythm (black)^13^. **b** Individual changes in the baseline of the glucose 24-h rhythm. Group means ± SD are indicated above each panel. **c** The peak time of the 24-h rhythm during night (blue) and day shifts (red). Blue star, mean peak time of night shifts; red star, mean peak time of day shifts; *p*-value derived from a Kuiper test. **d** Individual changes in the mean glucose response heights after meal consumption. **e**, **f** An individual example (ID 61) of continuous glucose monitor (CGM) data and the corresponding model fit during day (**e**) and night shifts (**f**). Blue, CGM data; orange, model prediction incorporating meal events and the underlying 24-h glucose rhythm; black, the underlying 24-h glucose rhythm; dashed lines, meal timestamps. *n* = 55 for all analyses that only included participants with available data from both CGM and MyFoodRepo smartphone app. See also Supplementary Table 5.

We next used a mathematical model of glucose levels (illustrated in Fig. 3a) that models glucose levels as a superposition of (1) meal-induced glucose spikes (orange lines) using the timestamped food and drink consumption entries recorded with the MyFoodRepo smartphone app (dotted gray lines), and (2) a 24-h rhythm in the underlying glucose levels (black line)^13^. The lowest point (i.e., the trough, nadir) of the baseline glucose levels was lower by −0.29 ± 0.69 mmol/L during night shifts compared to day shifts for the same study participants (*p* = 0.003, Fig. 3b). Furthermore, the 24-h baseline glucose levels were significantly shifted during night shifts (*p* < 0.001), with the peak time during afternoon on day shifts (red star, Fig. 3c) and late evening during night shifts (blue star, Fig. 3c). The model also revealed that the mean meal height (i.e., the glucose increase following a meal) was 0.23 ± 0.38 mmol/L higher during night compared to day shifts (*p* < 0.001, Fig. 3d). These differences are illustrated in one example, who exhibits a lower trough in glucose levels but higher average postprandial glucose spikes during night shifts compared to day shifts (Fig. 3f vs. Fig. 3e, respectively).

### A higher protein diet and the avoidance of nighttime eating are associated with lower glycemic variability in shift workers

**Fig. 4:**
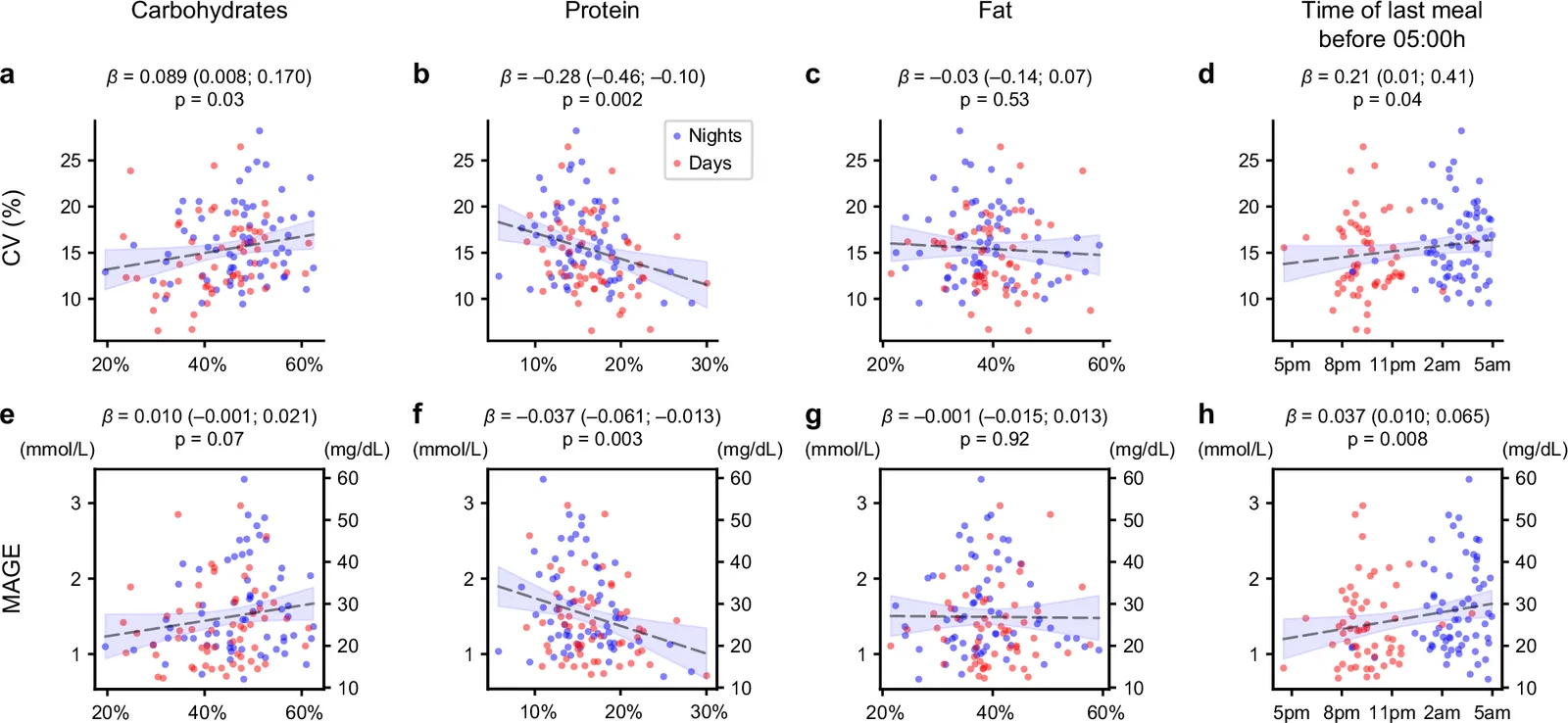
Glycemic variability is associated with increased carbohydrates, lower protein, and nighttime eating. In a linear mixed model pooling day (*n* = 59, red dots) and night shifts (*n* = 67, blue dots), glucose coefficient of variation (CV) and mean amplitude of glycemic excursions (MAGE)^16^ were associated with macronutrient proportions (CV: panels **a**–**c**; MAGE: panels **e**–**g**) and nighttime eating (panels **d** and **h**, respectively). β denotes the inferred regression coefficient (with a 95% confidence interval), which is also shown graphically (gray line and shaded blue, respectively).

To explore potential nutritional interventions in the shift worker population, we quantified the relationships between the macronutrient composition and nighttime eating with markers of glycemic variability, pooled across both shift conditions. Among the macronutrients, a higher proportion of carbohydrates was associated with a higher glucose CV (*p* = 0.03, Fig. 4a). Conversely, a higher protein intake correlated with reduced glucose fluctuations, reflected by a lower CV (*p* = 0.002, Fig. 4b) and decreased MAGE (*p* = 0.003, Fig. 4f). Fat intake percentage was not significantly associated with CV (*p* = 0.53, Fig. 4c) or MAGE (*p* = 0.92, Fig. 4g). To explore the correlation of nighttime eating with glycemic variability, we defined a continuous parameter as the time of the last calorie intake before 05:00 h (5 a.m.) (Supplementary Fig. 4) and found that those who ate throughout the night exhibited higher CV (*p* = 0.037, Fig. 4d) and MAGE (*p* = 0.008, Fig. 4h). To put these exploratory relationships in a clinical context, we hypothesize that an intervention targeting a 30% protein intake could reduce average night-shift MAGE levels from 1.59 to 0.98 mmol/L, and an intervention restricting eating between 20:00 h and 05:00 h would be expected to reduce night-shift MAGE from 1.59 to 1.33 mmol/L.

### Lower heart rate and plasma cortisol in the morning following night shifts

For the secondary outcomes, the anthropometric, cardiometabolic, and self-reported physical activity parameters were compared between the two series of shifts in Table 2. We found no differences in body weight, BMI or diastolic BP. However, higher morning systolic BP (+ 1.9 ± 6.9 mmHg, *p* = 0.03), but lower HR (−3.5 ± 10.0 beats/min, *p* = 0.005) and plasma cortisol (−108 nmol/L, IQR −210 to 33, *p* = 0.007) were observed post-night shift compared to the morning of day shift. After adjustment for multiple comparisons, the reductions in HR and plasma cortisol remained significant, while the increase in morning systolic BP was no longer significant (Table 2). There were no significant differences in other cardiometabolic parameters or self-reported physical activity between the two series of shifts.

**Table 2:** Comparison of cardiometabolic parameters and health outcomes between night and day shifts

Median (IQR)	*n*	Day shifts	Night shifts	Difference	*p*-value	Adjusted *p*-value
*Clinical data*						
Body weight (kg)	70	64.7 (54.3; 75.2)	64.4 (53.6; 75.0)	−0.2 (−1.1; 0.5)	0.08	0.22
Body mass index (kg/m^2^)	70	22.8 (20.6; 25.6)	22.8 (20.3; 26.2)	−0.1 (−0.4; 0.2)	0.055	0.17
Blood pressure^a^ (mmHg), mean ± SD						
Systolic	70 mmHg	111.3 ± 9.7	113.2 ± 8.8	1.9 ± 6.9	0.03	0.10
Diastolic		71.4 ± 7.4	72.8 ± 7.4	1.4 ± 6.0	0.052	0.17
Heart rate^a^ (beats/min), mean ± SD	69	70.7 ± 9.3	67.2 ± 11.3	−3.5 ± 10.0	0.005	0.02
Morning lab results						
Random plasma glucose^b^ (mmol/L)	68	4.9 (4.6; 5.2)	4.9 (4.5; 5.1)	0.0 (−0.3; 0.2)	0.68	0.85
Glycated hemoglobin (%)	68	5.2 (5.0; 5.3)	5.2 (5.0; 5.3)	0.0 (−0.1; 0.1)	0.98	0.92
Aspartate transaminase (U/I)	68	19.0 (15.0; 24.0)	19.0 (15.0; 22.5)	0.0 (−3.0; 3.0)	0.66	0.86
Alanine transaminase (U/I)	68	17.0 (12.5; 22.5)	17.0 (13.0; 22.5)	0.0 (−3.0; 2.0)	0.78	0.81
Gamma-glutamyl transferase (U/I)	68	13.5 (9.5; 17.5)	14.0 (10.5; 18.0)	1.0 (−1.0; 2.0)	0.19	0.43
Alkaline phosphatase (U/I)	68	49.5 (40.0; 56.0)	51.5 (43.5; 59.0)	2.0 (−2.0; 6.5)	0.002	0.01
Creatinine (µmol/l)	68	68.0 (60.0; 75.0)	68.0 (61.0; 74.5)	−0.5 (−4.5; 3.0)	0.55	0.79
Total cholesterol (mmol/L)	68	4.37 (3.98; 5.12)	4.50 (4.04; 5.20)	0.00 (−0.23; 0.39)	0.51	0.78
Low-density lipoprotein cholesterol (mmol/L)	68	2.38 (1.92; 2.94)	2.45 (2.07; 2.98)	0.06 (−0.22; 0.41)	0.19	0.38
High-density lipoprotein cholesterol (mmol/L)	68	1.60 (1.42; 1.80)	1.59 (1.33; 1.85)	−0.02 (−0.15; 0.12)	0.57	0.77
Triglycerides (mmol/L)	68	0.74 (0.56; 0.99)	0.67 (0.55; 0.87)	−0.06 (−0.15; 0.08)	0.11	0.29
Cortisol (nmol/L)	68	362 (288; 482)	306 (233; 393)	−109 (−210; 33)	0.0001	0.002
Thyroid-stimulating hormone (mIU/L)	68	1.50 (0.98; 2.12)	1.92 (1.56; 2.60)	0.43 (0.05; 0.93)	< 0.0001	< 0.003
*Physical activity, cognitive performance, mental health and well-being*						
Physical activity^c^ (IPAQ, MET-minutes/week)	70					
Low		1114 (487; 2822)	1201 (371; 4158)	38 (−2327; 941)	0.76	0.81
Moderate		240 (80; 720)	240 (0; 600)	0 (−160; 240)	0.44	0.70
High		960 (0; 1920)	840 (240; 1920)	0 (−480; 630)	0.71	0.85
Total		3227 (1866; 4893)	3231 (1782; 5724)	240 (−1745; 1287)	0.71	0.82
Sleepiness^d^ (SSS total score)	69	2 (1; 3)	4 (3; 4)	2 (0; 2)	< 0.001	< 0.01
SART	69					
Mean accuracy (%)		97.8 (95.1; 99.1)	96.9 (94.2; 98.2)	−0.4 (−2.2; 0.4)	< 0.001	< 0.008
Commission errors (%)		20 (8; 36)	24 (12; 40)	4 (−4; 16)	0.001	0.006
Omission errors (%)		0 (0; 1.0)	0 (0; 1.0)	0 (0; 0.5)	0.33	0.58
Reaction time (ms)		127.3 (94.6; 199.3)	127.1 (98.8; 183.6)	8.8 (−25.5; 32.9)	0.28	0.53
Inverse efficiency (ms)		131.2 (99.0; 200.1)	136.3 (103.3; 196.7)	11.9 (−25.7; 32.8)	0.19	0.41
Depression^e^ (PHQ-9 total score)	70	5 (2; 10)	5 (3; 9)	0 (−2; 2)	0.85	0.82
PHQ-9 depression severity, *n* (%)						
Not at all		6 (8.6)	7 (10.0)			
Minimal		28 (40.0)	26 (37.1)			
Mild		18 (25.7)	22 (31.4)			
Moderate		10 (14.3)	13 (18.6)			
Moderately severe		5 (7.1)	1 (1.4)			
Severe		3 (4.3)	1 (1.4)			
Well-being^f^ (WHO-5 index)	70	60 (52; 76)	60 (44; 72)	−2 (−12; 12)	0.43	0.71

*IPAQ* International Physical Activity Questionnaire, *MET* Metabolic Equivalent, *PHQ-9* Patient Health Questionnaire-9, *SSS* Stanford Sleepiness Scale, *SART* Sustained Attention to Response Task^17^, *WHO-5* WHO-5 Well-being Index.

^a^To account for circadian variation, values were compared between readings undertaken at the same time in the day (i.e., in the morning prior to the start of day shift rotation versus in the morning following the end of night shift rotation).

^b^One shift worker had a fasting plasma glucose within the prediabetes range, as defined by the American Diabetes Association^77^.

^c^Physical activity assessed with the short form of the International Physical Activity Questionnaire (IPAQ)^74^, with a total score and three subscores for low, moderate, and high physical activity levels.

^d^Sleepiness assessed with the Stanford Sleepiness Scale (SSS)^70^ on a 7-point scale, 1 representing feeling alert and 7 indicating being almost in reverie.

^e^Depression assessed with the Patient Health Questionnaire-9 (PHQ-9)^76^, score ranging between minimal (1–4 points), mild (5–9 points), moderate (10–14 points), moderately severe (15–19 points), and severe depression (20–27 points).

^f^Well-being assessed with the WHO-5 index^75^ ranging from 0 to 100; where 0 represents the worst possible and 100 the best possible quality of life.

### Night shifts are associated with time-of-day specific differences in heart rate and parasympathetic activation rhythms

**Fig. 5:**
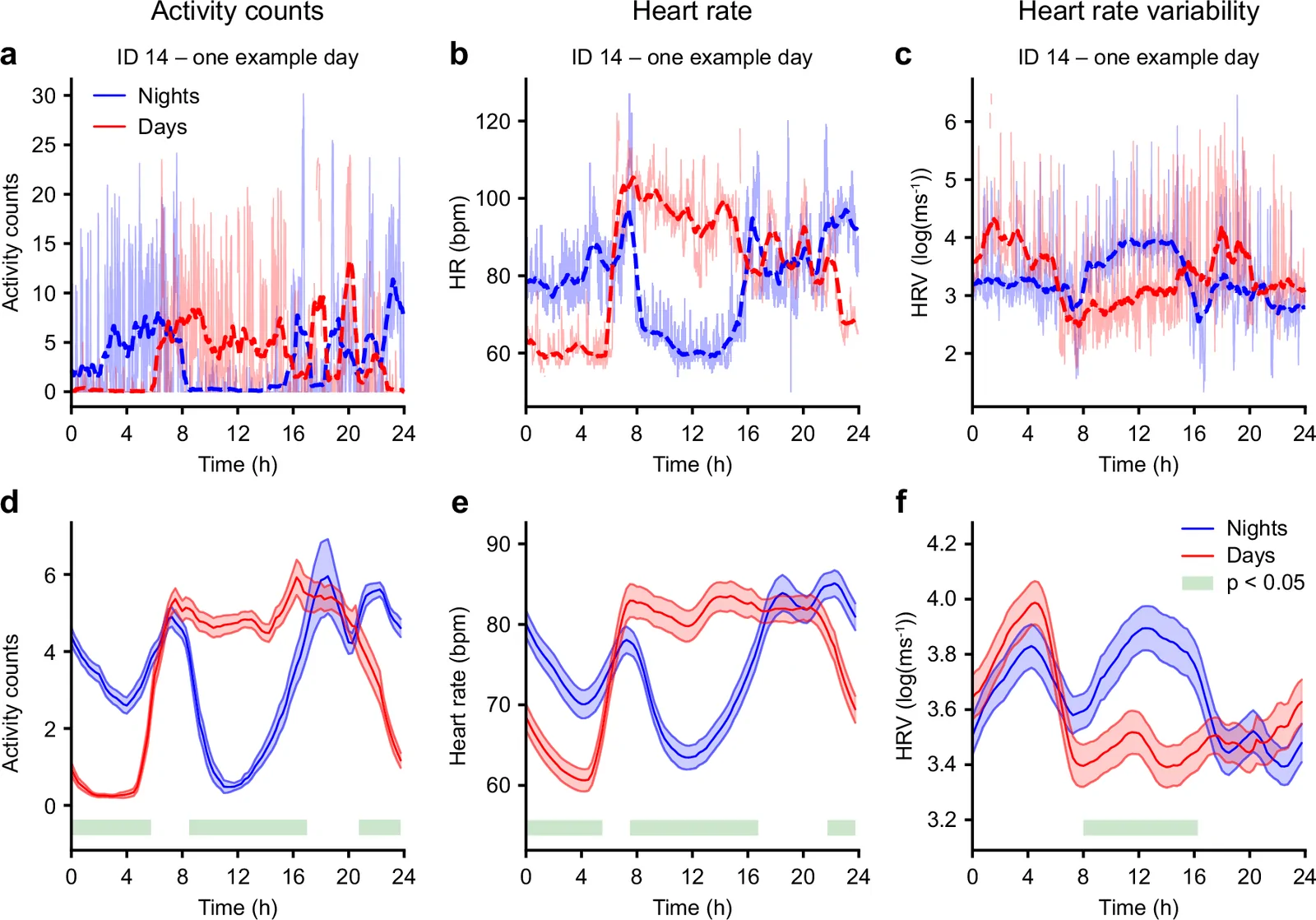
The mean 24-h profiles of activity counts (in arbitrary units), heart rate (HR, beats per minute, bpm), and heart rate variability (HRV) are altered during night shifts. **a**–**c** Examples from participant ID 14 of one day of measurements for activity counts, HR, and HRV, respectively. Blue, night shifts; red, day shifts. One-hour moving average estimates are shown as dashed lines. **d**–**f** The 2-h moving average over the 24-h clock of activity counts, HR, and HRV (log(RMSSD)), respectively. The shaded area represents the mean ± SEM, and the green bar represents significant group differences as assessed from *p* < 0.05 in a Wilcoxon non-parametric test, adjusted for multiple testing with the Benjamini-Hochberg procedure. *n* = 43 for all analyses. See also Supplementary Table 5.

The activity monitor and electrocardiogram ActiHeart^®^ provided continuous measurements around the 24-h clock for physical activity, HR, and heart rate variability (HRV) (examples of one day of measurements shown for one participant in Fig. 5a–c). Continuous actigraphy monitoring showed significantly modified activity profiles across 24 h, with night shifts leading to higher nighttime and lower daytime activity levels compared to day shifts (Fig. 5d). Activity levels were similar during the early morning (06:00 h to 08:00 h) and late afternoon/evening (17:00 h to 22:00 h) periods. The continuous HR data revealed significant disruptions in 24-h HR fluctuations (Fig. 5e, Supplementary Table 5). The changes to HR showed an overall similar pattern as activity counts (Fig. 5d) but with one notable difference: while the activity levels were comparable between night and day shifts at 08:00 h, the HR was lower during night shifts. This low morning HR in night shifts corroborates the independent morning measurement of resting HR with an arm-cuff BP monitor (see above, Table 2). In parallel, we also measured HRV, which reflects parasympathetic (vagal) activity and autonomic nervous system regulation. No significant difference was observed in the global HRV between shift periods (Supplementary Table 5). However, daytime HRV was significantly higher around midday on night shifts compared to day shifts (Fig. 5f).

### Night shifts led to poorer cognitive performance and sleep quality with higher levels of fatigue

As part of the secondary outcomes, cognitive performance was compared between night and day shifts using the Sustained Attention to Response Task (SART)^17^ at the end of both shifts (Table 2). Night shift work was associated with lower mean accuracy (−0.4%, IQR −2.2 to 0.4, *p* < 0.001) and higher commission errors (4%, IQR −4 to 16, *p* = 0.001) compared to day shifts. However, we found no significant differences in omission errors, reaction time, and inverse efficiency. Higher levels of sleepiness were reported post-night shift compared to post-day shift (+2 on a 7-point scale, IQR 0 to 2, *p* < 0.001) just before performing SART. The differences in mean accuracy, commission errors, and levels of sleepiness remained significant after adjustment for multiple comparisons (Table 2).

Self-reported sleep and fatigue between night and day shifts are compared in Supplementary Table 6. Shift work schedules significantly affected the timing of the main sleep-wake pattern (both *p* < 0.001), and participants reported taking more naps during night shifts compared to day shifts (*p* < 0.001). Poorer sleep quality was more commonly reported during night shifts compared to day shifts, as well as insufficient sleep, inadequate restfulness after sleep, and waking earlier than intended (all *p* < 0.001). In our previous analysis of sleep diaries of the same cohort^14^, sleep duration (i.e., main sleep period) remained stable across day shifts, whereas during night shifts, sleep duration was significantly longer prior to the first night shift but shorter on subsequent night shifts (all *p* < 0.001). We observed similar trends for sleep quality between night and day shifts^14^. In terms of fatigue, a higher proportion of participants reported feeling tired during night shifts compared to day shifts (*p* < 0.001).

### Higher emotional stress but lighter time pressures were perceived during night shifts

As part of the secondary outcomes, levels of self-reported mental health and well-being were all similar between night and day shifts (all *p* > 0.05, Table 2). We used a modified version of the Standard Shiftwork Index^18^ to explore physical and mental workloads of shift workers. In general, most participants stated that they were about the same between night and day shifts. However, emotional stress was more often perceived as higher during night shifts, while time pressures were generally considered lighter during night shifts (Supplementary Table 2).

## Discussion

This study provides a comprehensive assessment of the short-term impact of shift work in real-world occupational environment on nutritional, cardiometabolic, behavioral, physiological, and psychological outcomes by utilizing multiple wearable devices, smartphone dietary app, clinical lab assays, and attention testing. A significant change was observed in the dietary behaviors of the same participants between night and day shift periods, particularly in caloric and macronutrient intake, as well as meal timing. These changes may have, in part, contributed to alterations in glucose metabolism during the night shift periods.

A recent meta-analysis concluded that there are no intraindividual differences in 24-h energy intake between day shifts and night shifts^19^. Of the seven included studies in a within-participant comparison, two found higher energy intake on night shifts^20,21^ and two others reported no difference^22,23^. Three studies reported lower intake on night shifts^24–26^, which are consistent with our findings. The mean difference in energy intake between shifts observed in our study (−163.8 kcal/day) is comparable to that reported by Bouillon-Minois et al. (−206 kcal/day)^26^. Work-related stress-induced food restriction has been suggested as a contributing factor to the reduced energy intake observed during night shifts^26^. Our findings support this notion, as shift workers in the present study reported lower subjective appetite scores and fewer meals consumed during night shifts compared to day shifts. Our macronutrient analysis showed that the lower energy intake during night shifts was due to reduced consumption of protein and fat, but not carbohydrates. This is in line with previous studies showing trends towards low protein^21,24–26^ and fat intake^25–27^ during night compared to day shifts. The availability and accessibility of high energy-dense snacks during night shifts explained the majority of energy intake that comes from carbohydrate consumption^28^. Previous studies showed lower intake of fruit and vegetables among night shift workers than day workers^29,30^, which is consistent with our findings of lower dietary fiber intake during night shifts compared to day shifts, but both were below the recommended 30 g of daily intake. The intake of fruit and vegetables should be encouraged in shift workers given the important role of dietary fiber for the prevention of cardiometabolic disease^31^.

While total energy and macronutrient intake in shift workers have been extensively studied^19^, the temporal distribution of calorie and macronutrient intake in relation to shift schedules remains largely underexplored^27,32^. In the present study, mealtimes shifted significantly during night shifts, with more calories and macronutrients consumed at night compared to day shifts, corroborating previous findings of higher nighttime calorie intake during night shifts compared to daytime schedule^27^. Indeed, following a night shift, shift workers spent the daytime sleeping^14^, which often led to skipping lunch. Our findings on self-reported eating habits supported this, as the majority of participants indicated that dinner became the main meal during night shifts, whereas lunch was the main meal during their day shifts. Additionally, participants reported that skipping meals, irregular eating patterns, and increased snacking were the most common dietary adjustments in response to the night shift schedule, consistent with previous qualitative research among shift workers^28^.

Both laboratory-controlled and epidemiological studies have demonstrated that night shift work (circadian misalignment) leads to increased fasting plasma glucose, impaired glucose tolerance, higher postprandial glucose concentrations, elevated insulin levels, and decreased insulin sensitivity^5,7,12,33,34^. Our study extends these findings by showing that night shift work contributes to increased glycemic variability with higher postprandial glucose spikes, relative to day shifts. This is important, as even in the absence of diabetes, higher glycemic variability is a risk factor for coronary atherosclerosis and may predict incident cardiovascular events and type 2 diabetes^35,36^. The biological mechanisms underlying shift work-induced impaired glucose regulation remain poorly understood, but several potential mechanisms have been proposed. Morris et al. demonstrated that in healthy adults undergoing a circadian misalignment protocol, lower glucose tolerance in the evening was mediated by reduced β-cell function and decreased insulin sensitivity^7^. In a later study involving circadian-misaligned adults, Wefers et al. found that decreased muscle insulin sensitivity resulted from reduced skeletal muscle non-oxidative glucose disposal. Furthermore, misalignment of the muscle molecular clock favors intramuscular fatty acid metabolism over glucose metabolism, leading to disrupted energy metabolism^12^. In another study, Sharma et al. found that the higher postprandial glucose concentrations observed during night shifts were due to impaired β-cell responsiveness to glucose^34^. Taken together, these findings may explain the higher glycemic variability and postprandial glucose spikes observed during night shift periods in our study.

In this study, we found that not only the timing of meals but also the composition of meals was associated with glycemic variability in a pooled analysis across both shift conditions. In particular, higher daily proportion of carbohydrate intake correlated with higher glycemic variability, whereas higher daily proportion of protein intake correlated with lower glycemic variability. However, these findings are hypothesis-generating and should not be interpreted as evidence that a high-protein dietary intervention would improve glycemic variability, as the observed associations cannot establish causality. These findings require confirmation in future controlled dietary intervention studies. Consuming proteins alongside carbohydrate-rich meals during the day can effectively lower postprandial blood glucose peaks^37,38^. Furthermore, consuming a high protein and low carbohydrate meal at night significantly reduced postprandial glucose excursions by 71.4%^39^. This effect is driven by enhanced insulin sensitivity, influenced by the insulinotropic properties of dietary proteins and slower gastric emptying^40^. Regarding meal timing, we found that nighttime eating (defined as consumption up until 05:00 h) was associated with high glycemic variability. Our study expands upon a previous observational study in shift workers, which found that eating at night (21:00 h to 06:00 h) led to significantly higher insulin levels compared to consuming most calories during the daytime (06:00 h to 21:00 h)^41^. Additionally, studies indicate that blood glucose levels after a meal are significantly higher when food is consumed at night compared to the same meal eaten in the morning^42–44^. When adjusting for shift condition, the confidence interval of the relationship between meal timing and MAGE widened. Given that night shift work and late-night eating are strongly correlated, their overlapping influence makes it difficult for statistical models to separate their individual effects using observational data. These limitations further reinforce the need for future controlled interventional trials to isolate the specific impact of targeted dietary strategies.

Both HR and HRV exhibit a day-night rhythm. HR increases during the day and decreases at night, whereas HRV shows the opposite pattern, decreasing during the day and increasing at night^45,46^. As shown by our data, these 24-h patterns of the autonomic nervous system were altered during night shifts. This imbalance in autonomic nervous system regulation could be explained by the changes in behavior factors during night shift periods, such as sleep, physical activity, and posture^47^. In a recent meta-analysis, shift workers exhibited elevated sympathetic nervous system activity, as suggested by reduced HRV^48^, which was not observed in our data. It remains to be investigated whether the observed alteration in autonomic nervous system regulation represents an adaptive response to night shift work or a maladaptive change that may contribute to adverse health outcomes.

Shift work affects cortisol secretion patterns^49^. Our observation of lower morning cortisol levels following night shifts, compared to mornings after non-night shifts, corroborates the findings reported by Ernst et al.^50^. It has been suggested that the depressed cortisol level post-night shifts may be associated with alterations in cortisol rhythm, with the timing of morning cortisol rise shifted to a later time in a day^50,51^. Approximately two days off work are required to restore the altered diurnal rhythm of cortisol secretion^52^, however, we did not measure cortisol during time off after night shifts in our study for logistic reasons. Our results should be interpreted with caution as differences in the interval between awakening and blood sampling may represent a potential confounding factor. Given the influence of the cortisol awakening response on morning cortisol levels^53^, variation in wake-to-sampling intervals between post-night and pre-day shift conditions may have contributed to the observed differences in cortisol levels.

Sleep deprivation and disturbances are prevalent among shift workers due to the imposed rhythms that are in desynchrony with their endogenous circadian clocks^3^. Expanding on our recent findings from the same cohort of shift workers^14^, we now report that night shift work is associated with poor sleep quality, insufficient sleep, inadequate restfulness, and waking earlier than intended. These findings are consistent with the earlier work by Boivin et al.^3^. Sleep disturbances have implications for job performance, as it leads to fatigue, impaired cognitive function, such as attention and executive performance, and burnout^11,54^. Indeed, we found increased levels of tiredness and decreased levels of attention, as demonstrated by the poorer performance on the SART, post-night shifts compared to post-day shifts. Several pharmacological and non-pharmacological interventions, such as melatonin use and napping strategies, have shown promise in reducing sleep disturbances among shift workers^55–57^. In the current cohort, approximately 15.3% of the participants reported using melatonin to aid sleep and more frequent napping during night shifts compared to day shifts.

The main strength of OPTI-SHIFT is its real-world setting, comparing the same participants’ 24-h behavioral and physiological patterns between the night and day shift periods, complementing findings from epidemiological and laboratory-controlled studies. The within-participant comparison also eliminates many confounders associated with interindividual variability in between-participant comparisons. Additionally, the homogeneity of the study population and setting (i.e., 96% of healthcare workers) reduced confounders associated with the heterogeneity of shift work patterns across various occupational settings. Also, the use of wearable devices for continuous measurements provided in-depth longitudinal high-resolution insights into 24-h changes in cardiometabolic parameters compared to a single time-point assessment. The use of the MyFoodRepo food diary app to assess temporal eating patterns throughout shift schedules that include timing of meals has significantly enhanced the accuracy of data collection, compared to routinely used self-reporting food diaries^20–26^.

This study has several limitations. First, the order of night and day shifts was not randomized, as such randomization is incompatible with a real-life setting, owing to operational constraints (predefined work schedules, frequent rota adjustments, staff availability, and sick leave), as well as the social and familial commitments of healthcare workers. Second, the study population consists of generally healthy young shift workers with normal BMI, despite our efforts to recruit participants with a diverse range of comorbidities and BMI categories. This limits the generalizability to other shift worker populations with different health statuses, professions, and occupational settings. Third, menstrual cycle phase, which may influence physiological and behavioral outcomes^58^, was not assessed during the study and could therefore not be included as a covariate in the analysis. Fourth, despite the homogeneity of the present cohort (i.e., 96% of healthcare workers), there remains a wide variability in job responsibility (physical and mental demands), years of exposure to night shift work, and shift work patterns (most often irregular vs. regular and permanent night shifts). All these factors have been shown to influence individual adaptation to circadian misalignment^3^. Fifth, the focus of this study is on the acute, short-term physiological effects of shift work. Follow-up studies designed to investigate its long-term cardiometabolic effects are required. Within our study, the total duration of shift work was highly correlated (Pearson coefficient ρ = 0.85) with age, a known cardiometabolic risk factor. Once age was adjusted for, we did not see a significant association between long-term shift work duration and any of the components of metabolic syndrome in our young and healthy population. Sixth, we did not measure BP and cortisol levels continuously throughout the day to limit the burden on participants, which would have provided a more comprehensive understanding of their variations and fluctuations over time. In addition, the majority of participants did not fast before the post-night shift blood tests; therefore, some lab results should be interpreted with this consideration in mind. Finally, data collection did not extend to after the series of night shifts, which could have provided more insights into the lasting effects of circadian misalignment on the measured parameters. The interval between the two shift periods ranged from weeks to several months for organizational reasons, thus occurring in different seasons, and might have biased the comparison between shifts: for day shift rotation, 47 were in summer and 23 in winter, and for night shift rotation, 32 were in summer and 40 in winter^14^. Reduced light exposure associated with seasonal variation has been linked to changes in sleep timing and duration, as well as lower mood^59,60^. Future studies should account for seasonal variation in light exposure, e.g., by balancing measurements across seasons if feasible, or adjusting for season of each measurement as a covariate in the analysis.

In conclusion, night shift work is associated with adverse cardiometabolic health and cognitive outcomes. There is an urgent need to implement lifestyle countermeasures to improve the health and well-being of shift workers. Our findings reported here suggest that modifying macronutrient intake and meal timing during night shift work may be beneficial. Further investigation through randomized controlled trials assessing these dietary strategies to improve the cardiometabolic health of night shift workers is now warranted.

## Methods

### Study design and participants

OPTI-SHIFT was a prospective, observational, single center study undertaken at Geneva University Hospitals (HUG), Switzerland. Participants were recruited via posters, announcements, oral presentations, social media, and the hospital website. All participants provided written informed consent prior to enrollment in the study. Data collection was conducted from February 2022 to August 2024. The study protocol was approved on 4 August 2021 by the Ethics Committee of the Canton of Geneva (BASEC no. 2021-01008) and registered on 15 December 2021 on ClinicalTrials.gov (no. NCT05177965). This study was carried out in accordance with the Declaration of Helsinki, the Good Clinical Practices and the Swiss Human Research Act.

Participants were assessed at two time points to compare the effects of a series of three consecutive night shifts (circadian misalignment) to a series of three consecutive day shifts (circadian alignment, Supplementary Fig. 1). Each time point consisted of two visits, and participants thus attended four study visits over the whole study. At each time point, the set-up visits (visits 1 and 3) occurred at least 24 h before a series of night or day shifts to ensure that measurements were accurate to cover the series of shifts, i.e., the accuracy of continuous glucose monitors (CGM) tends to improve 12–24 h after insertion. The follow-up visits (visits 2 and 4) took place after a series of night shifts and after a series of day shifts, respectively. The second time point was scheduled at least two weeks after the last night shift or return from travel across two or more time zones, to ensure that circadian misalignment period had ended (i.e., that the circadian clocks had been realigned). The interval between the first and second time points was kept ideally under three months, for feasibility and to reduce the effects of daylight and seasons on measurements.

### Inclusion and exclusion criteria

#### Inclusion criteria

- Clinical criteria
  - Men and women
  - Age of 20–50 years (to avoid too much variability in the circadian clock parameters)

- Work-related criteria
  - Working ≥80% full-time equivalent over the previous month and during the study
  - Working night shifts (i.e., shifts that comprise working hours between 23:00 h and 06:00 h) in rotation with day shifts (i.e., shifts that comprise working hours between 06:00 h and 20:00 h)
  - Planned to work at least three consecutive night shifts during the study preceded by at least a day off or day shift
  - Planned to work at least three consecutive day shifts during the study

- Confident use of a smartphone compatible with the study application (iOS, Android) and able to regularly take pictures of consumed food/drinks
- Able to give fully informed consent and follow the study procedures

#### Exclusion criteria

- Clinical criteria
  - Planned or current pregnancy during the study
  - Menopausal women (due to changes in sleeping habits, hormonal status, and sometimes the impact on weight and mental concentration)
  - Major illness or hospitalization over the previous month
  - Carries a pacemaker, pump, or other medical devices that can be disabled by a magnet
  - Major mental illness

- Study-related criteria
  - Trip to a different time zone (≥2-h time difference) over the previous month or planned during the study
  - Enrolled in an interventional clinical trial (potentially interfering with the main outcomes) over the previous month or planned during the study

### Outcomes

#### Body composition and vital signs

Weight was measured in light clothing, and the body mass index (BMI) was calculated as the weight in kilograms divided by the height in meters squared (Supplementary Fig. 1). Fat mass and fat-free mass were estimated from the electric resistance and reactance measured by BIA using the Nutriguard-M^®^ device (Data Input, Germany). Skin adhesive electrodes were placed on the right hand and foot, while the subject was lying on their back. An alternating electrical current of 50 kHz and 0.8 mA was applied. The measured resistance and reactance were used to estimate fat-free mass by a formula developed in Geneva and validated against dual energy X-ray absorptiometry^61^. Fat mass was calculated by subtracting fat-free mass to weight. Fat mass index and fat-free mass index were calculated by dividing them by the height squared. Waist and hip circumferences and waist-to-hip ratio (WHR) were assessed according to the WHO standard procedure^62^. BP was measured three times with a calibrated monitor (Omron Intellisense BP monitor, Omron Healthcare, USA) and an appropriately sized arm cuff, after 5 min of rest in the sitting position, and the last two values were averaged.

#### Blood measurements

Fasting plasma glucose, glycated hemoglobin (HbA1c), lipid profile (total, HDL and LDL cholesterol, triglycerides), liver function tests, cortisol, and full blood count were measured with the routine assays at the clinical lab of HUG. Blood draw at the end of the night shifts could also be performed in the non-fasting state to reduce the burden to study participants.

#### MyFoodRepo smartphone application

The MyFoodRepo smartphone food diary app (Digital Epidemiology Lab, EPFL) recorded the timestamps of consumed food and drink along with pictures, barcodes of packaged items, or free text descriptions^63^. The accuracy of this food diary app has been validated against reference values from weighed food diaries^64,65^. A minimum number of 2–3 days required to obtain reliable estimates of dietary intake has been established from a large digital cohort^66^. This app has been used as a dietary assessment tool in nutrition studies^65,67^. The macronutrient composition of each consumed item was automatically extracted from a constantly updated nutritional composition table, verified by trained dieticians.

#### Continuous glucose monitors (CGM)

CGM data was recorded with an Abbott FreeStyle Libre^®^ Pro device (Abbott Diabetes), which reports interstitial glucose every 15 mins. During the initial period of recording, the CGM data can occasionally spend the majority of the time at the device minimal value of 2.2 mmol/L (40 mg/dL) due to the transient effects of microtrauma, local pressure leading to lower blood flow and device calibration, and in this case, we removed the initial data until the device exited this floor state after visual inspection of CGM raw data. As a preliminary step of glucose dynamics modeling (see below), abnormally low glucose values (that are typical of the early recording hours) were also corrected by removing long timescale trends within the time series. CGM metrics related to glycemic control were extracted from the CGM data using the R package ‘rGV’, and we focused on the mean glucose level, coefficient of variation (CV = SD/mean), and the mean amplitude of glycemic excursions (MAGE)^16^.

#### Chest-worn activity and heart monitor

The CamNTech ActiHeart^®^ device (version 5, CamNTech, United Kingdom) assessed physical activity (activity counts, proprietary algorithm), heart rate (HR, beats/min), and heart rate variability (HRV, using log root mean square of successive differences [log(RMSSD)] between normal heartbeats)^68^.

#### Sustained attention to response test (SART)

Cognitive performance was assessed using the Sustained Attention to Response Task (SART)^17^ on the online platform PsyToolkit^69^, right after the SSS questionnaire^70^ (see below, Supplementary Fig. 1). This is a 5-min standard test undertaken where participants were seated in front of a computer screen in a quiet room of the Clinical Trial Unit. After a short warm-up session, participants were tested individually and remained alone in the room, with no interaction or human contact throughout the task. No interruptions were permitted during the assessment. Participants were presented with an integer between 1 and 9, appearing 225 times in random order with different sizes and asked to press a key when they saw a digit on screen, but withheld it when they were presented with the number ‘3’, thus testing reaction time and inhibition. Outcomes are reported as mean accuracy, commission errors (a key was pressed when no key should be pressed, i.e., after a ‘3’, the ‘NO-GO trials’), omission errors (lack of key press when it should have been pressed, i.e., after a digit other than a ‘3’, the ‘GO trial’), reaction time (ranging between 0 and 1500 milliseconds; where lower values indicate better performance), and inverse efficiency (a combined measure of speed and accuracy with a higher score indicates poorer performance).

#### Questionnaires

We recorded socio-demographic data (e.g., age, sex, ethnicity), and work-related variables (e.g., work pattern, shift work system, motivations, workload) using a subset of questions adapted from the Standard Shiftwork Index^18^. We used the REDCap platform for data entry and questionnaires^71^. We assessed the self-reported sleep duration, sleeping times, and sleep quality using the Pittsburgh Sleep Quality Index (PSQI)^72^. The PSQI has seven components (subjective sleep quality, sleep latency, sleep duration, sleep efficiency, sleep disturbances, use of sleeping medications, and daytime dysfunction), with each component score ranging from 0 to 3, with the total score ranging from 0 to 21, where a higher score indicates poorer sleep quality. The chronotype was assessed using the Morningness-Eveningness Questionnaire (MEQ), with scores ranging from ‘definitely morning’ (70–86 points), ‘moderately morning’ (59–69 points), ‘neutral’ (42–58 points), ‘moderately evening’ (31–41 points), to ‘definitely evening’ (16–30 points)^73^. Physical activity levels were assessed using the short form of the International Physical Activity Questionnaire, with a total score and three subscores for low, moderate, and high physical activity levels^74^. Well-being was evaluated with the World Health Organization-Five Well-being Index with scores ranging from 0 to 100; where 0 represents the worst possible, whereas 100 represents the best possible quality of life^75^. Mental health was evaluated by the Patient Health Questionnaire-9, with scores ranging from minimal (1–4 points), mild (5–9 points), moderate (10–14 points), moderately severe (15–19 points), and severe (20–27 points) depression^76^. The Stanford Sleepiness Scale (SSS) evaluates the level of sleepiness at a specific time in the day that uses a 7-point scale, 1 representing feeling alert and 7 indicating being almost in reverie^70^. Self-reported eating habits were collected using a questionnaire and subjective appetite was measured using a visual analog scale ranging from 0 (not hungry) at all to 10 (very hungry).

### Statistics

#### Calculating 24-h physiological profiles using a 2-h rolling average

For the nutritional variables, the mean consumption rate per hour was calculated by using a kernel density estimate (with a von Mises distribution, *κ* = 30) and then normalizing such that the total area under the curve equaled the daily consumption rate. To calculate the moving average 24-h profile for glucose, activity counts, HR, and HRV, we first defined grid points spanning from 0 to 24 h every 15 min. We then computed the average values over a 2-h window centered on each grid point. For glucose, the common definition of coefficient of variation (CV = SD/mean) is calculated over all data points. We thus used this definition to calculate the 2-h moving average CV by dividing the 2-h moving average standard deviation by the 2-h moving average mean at each grid point.

#### Modeling ingestion events and glucose data

We combined the ingestion events with CGM data based on a recently published computational framework^13^. The food and drink consumption entries recorded with the MyFoodRepo smartphone app are used to model postprandial interstitial glucose increases, and both the height of the glucose spikes (termed “response heights”) and the time needed for glucose to return to baseline (termed “response half-life”) are inferred for each individual and each shift condition independently. The model also includes an underlying 24-h cosinor function to describe 24-h oscillations in baseline (i.e., irrespective of meal intake) glucose levels, which is a mathematical model that assumes baseline glucose levels follow a sinusoidal pattern over the course of a day. This 24-h cosinor function is described by the three parameters ‘baseline’, ‘peak-to-trough amplitude’, and ‘peak time’, which are similarly learned for each participant and each shift condition independently. The model was trained by minimizing the error between the model and the CGM data using a maximum a posteriori probability (MAP) estimate from 200 different starting conditions, and the settings and parameters of the original method were left unchanged.

#### Missing data handling

The MyFoodRepo smartphone app data were considered missing when fewer than 700 calories per day were recorded (Supplementary Fig. 5). The threshold of 700 calories was chosen empirically based on manual inspection of the MyFoodRepo and CGM data showing multiple glucose spikes without corresponding meal entries below this threshold. The CGM metrics were calculated when at least 48 h of data were recorded. We calculated metrics from the first 48 h of the MyFoodRepo and CGM data to ensure a comparable measurement window between day and night shifts and to avoid oversampling of certain times of the day in either group. The MyFoodRepo data were analyzed alongside the CGM data when both MyFoodRepo data and CGM data were concomitant and not missing. Upon data extraction, the ActiHeart^®^ produces a quality estimation of the signal (range 0–1), and we filtered the data based on the threshold of 0.8. The ActiHeart^®^ data was used when at least 2000 valid 1-min time points were recorded, and the 2-h rolling average was considered missing when fewer than 10 valid measurements were available for each 2-h window.

#### Statistical analyses

Continuous and normally distributed variables are reported as mean ± SD and compared with paired *t*-tests using the matched day and night data for each participant. In this within-participant comparison, we thus did not adjust for variables such as age, sex, body composition, and physical activity. Normality was assessed with the Shapiro-Wilk test. Non-normally distributed continuous variables are reported as median (IQR) and compared with the Mann–Whitney U test (i.e., Wilcoxon rank-sum test). Categorical variables are presented as the number of participants (% in group) and compared using the chi-squared test.

Associations between the nutritional and glycemic variables were quantified by combining the day and night shift data within a linear mixed regression model accounting for repeated data in individual participants, where the glycemic variables were considered as the dependent variables and the nutritional variables (calories, macronutrient proportions, and nighttime eating) were considered as the independent variables. The aim of the random effect was to account for potential correlations within the same participant, and the model was fitted using the ‘MixedLM’ class from the ‘statsmodels’ Python library (v0.13.0). For each outcome, two models were fitted: (1) an unadjusted model omitting shift work, and (2) an adjusted model incorporating shift work as a covariate. We performed a complete-case analysis using all available data and hence no imputation methods were used for missing data. To account for multiple comparisons, *p*-values for secondary outcomes were adjusted using the Benjamini–Hochberg procedure to control the false discovery rate. As this was an exploratory observational study, the sample size was not predetermined and multiple testing corrections were not performed, unless stated otherwise. Nevertheless, we aimed to include a large sample of individuals to maintain statistical power, allowing for a sufficient margin to account for invalid data due to compliance and technological issues commonly encountered in observational studies involving wearable devices and apps.

## Supplementary information

OPTI-SHIFT Supplementary information.2026.08.22 (clean)

## Data Availability

The datasets generated and analyzed during the current study are not publicly available due to data privacy reasons, but are available from the corresponding author on reasonable request.

## Code availability

The underlying code for this study is not publicly available, but may be made available to qualified academic researchers on reasonable request from the corresponding author.
