# Supplementary material for: The nutritional and metabolic impact of night shift work in a real-world setting
Source: NPJ Biol Timing Sleep. 2026 Sep 29;3:44. doi: 10.1038/s44323-026-00107-y (PMC13623919; doi:10.1038/s44323-026-00107-y)
Supplement: Supplementary file 1 [file 44323_2026_107_MOESM1_ESM.pdf]

SUPPLEMENTARY INFORMATION

Supplementary Table 1. Participant characteristics (*related to Table 1*). 2
Supplementary Table 2. Work patterns, shift work systems, motivations, and workload. 4
Supplementary Table 3. Comparison of calorie and macronutrient intake between night and day shifts (*related to Figure 1*). 6
Supplementary Table 4. Self-reported eating habits, alcohol and caffeine intake, and tobacco use between night and day shifts. 7
Supplementary Table 5. Comparison of CGM metrics, actigraphy measurements and fasting insulin between night and day shifts (*related to Figures 2-3-5*). 8
Supplementary Table 6. Comparison of sleep and fatigue between night and day shifts (*related to Table 2*). 10

Supplementary Figure 1. Study design of the OPTI-SHIFT observational study. 12
Supplementary Figure 2. Flow diagram of study participants from screening, assessment of inclusion/exclusion criteria, enrollment, measurements, to data analysis..13
Supplementary Figure 3. Temporal distribution of fiber and alcohol consumption during day and night shifts (*related to Figure 1*). 14
Supplementary Figure 4. Temporal distribution of ingestion events during day and night shifts. 15
Supplementary Figure 5. Distribution of daily calorie intake recorded with the MyFoodRepo smartphone app over all shifts and participants of the study. 16

Supplementary Table 1. Participant characteristics (*related to Table 1*).

**Participant characteristics**	***n* = 72**
Educational level, *n* (%)	
College, High school	3 (4.2)
Apprenticeship	5 (6.9)
Professional school	39 (54.1)
University, University of applied science	21 (29.2)
Other	4 (5.6)
Household composition, median (IQR)	
Number of children	0 (0; 1)
Age of youngest child, years	3.5 (1.5; 7.0)
Age of oldest child, years	6.0 (3.6; 12.3)
Number of older incumbent persons	0 (0; 0)
Self-reported ethnicity, *n* (%)	
African	5 (6.9)
European	65 (90.3)
North American	2 (2.8)
South American	3 (4.2)
Other ethnicity	2 (2.8)
**Clinical data**	
Body mass index categories, *n* (%)	
Underweight	2 (2.7)
Normal	49 (68.1)
Overweight	16 (22.2)
Obesity	5 (7.0)
Self-reported comorbid conditions, *n* (%)	
Hypertension	1 (1.4)
Dyslipidemia	3 (4.2)
Obstructive sleep apnea	1 (1.4)
Thyroid disorder	3 (4.2)
Asthma	9 (12.5)
Cancer	2 (2.8)
Depression	3 (4.2)
Self-reported regular medication, *n* (%)	
Contraceptive pill, or intrauterine device	27 (37.5)
Painkillers, or muscle relaxant drugs	6 (8.3)
Asthma inhalers, or anti-histaminic drugs	4 (5.6)
Anti-depressant drugs	3 (4.2)
Thyroid hormones	2 (2.8)
Other medications, vitamins, supplements	8 (11.1)
Smoking status, *n* (%)	
Current	11 (15.3)
Past	11 (15.3)
Never	50 (69.4%)

**Participant characteristics**	***n* = 72**
Chronotype[1] (*n* = 69), *n* (%)	
Definitely morning type	0
Moderately morning type	21 (30.4)
Neutral type	43 (62.3)
Moderately evening type	5 (7.3)
Definitely evening type	0

Footnotes: [1]The chronotype was assessed with the Morningness-Eveningness Questionnaire, with a total score ranging between 'definitely morning' (70–86 points), 'moderately morning' (59–69 points), 'neutral' (42–58 points), 'moderately evening' (31–41 points), and 'definitely evening' types (16–30 points)[1].

Supplementary Table 2. Work patterns, shift work systems, motivations, and workload.

**Shift work schedule**	***n* = 72**
**Employment**	
Work time, hours	
Weekly work duration excluding overtime	40.0 (35.5; 43.3)
Weekly overtime	1 (0; 2)
Commuting time, minutes	
Day shift to work	20 (15; 30)
Day shift from work	20 (15; 30)
Night shift to work	15 (10; 30)
Night shift from work	20 (15; 30)
**Shift work system**	
No. successive day shifts, days	3.0 (2.5; 4.0)
No. successive night shifts, days	3.0 (2.5; 3.5)
No. successive number of days off	2.0 (2.0; 2.5)
Maximum no. successive shifts of any kind between days off in the past month	5 (4; 5)
No. weekends off per month	2 (2; 2)
No. night shifts in a year, days	55 (36; 72)
Types of night shifts (*n* = 70), *n* (%)	
Permanent night shift	1 (1.4)
A single block of night duty per year	1 (1.4)
Occasional blocks of night duty per year	9 (12.7)
A block of nights per month	11 (15.5)
Two blocks of nights per month	23 (32.4)
One or two nights each week	5 (7.0)
Other	21 (29.6)
**Impact of shift work on socio-familial life**	
Main reason for working shifts, *n* (%)	
Part of the job	70 (97.2)
The only job available	2 (2.8)
More convenient for domestic responsibility	4 (5.6)
Higher rates of pay	4 (5.6)
Others	8 (11.1)
Willingness to give up working shifts over day time job without shifts, *n* (%)	
Definitely not	6 (8.3)
Probably not	12 (16.7)
Maybe not	7 (9.7)
Probably yes	25 (34.7)
Definitely yes	22 (30.6)
Partner's weekly paid employment (*n* = 47), hours	42 (40; 42)

**Shift work schedule**	***n* = 72**
Partner's usual work pattern, *n* (%)	
Daytime - no shifts	30 (63.8)
Rotating shifts with nights	10 (21.3)
Rotating shifts without nights	2 (4.3)
Permanent night shifts	2 (4.3)
Other	3 (6.4)
Partner's perception of participants' work shifts, *n* (%)	
Extremely unsupportive	2 (4.3)
Fairly unsupportive	3 (6.4)
Quite indifferent	6 (12.8)
Fairly supportive	16 (34.0)
Extremely supportive	20 (42.6)
**Self-rated workload during night shifts compared to day shifts**	
Physical workload, *n* (%)	
Much lighter	9 (12.5)
A bit lighter	18 (25.0)
About the same	28 (38.9)
A bit heavier	8 (11.1)
Much heavier	9 (12.5)
Mental workload, *n* (%)	
Much lighter	5 (6.9)
A bit lighter	20 (27.8)
About the same	24 (33.3)
A bit heavier	13 (18.1)
Much heavier	10 (13.9)
Time pressures, *n* (%)	
Much lighter	20 (27.8)
A bit lighter	23 (31.9)
About the same	13 (18.1)
A bit heavier	11 (15.3)
Much heavier	5 (6.9)
Emotional stress, *n* (%)	
Much lighter	6 (8.3)
A bit lighter	15 (20.8)
About the same	19 (26.4)
A bit heavier	23 (31.9)
Much heavier	9 (12.5)

Footnotes: Questions adapted from a subset of questions in the Standard Shiftwork Index[2]. Data are presented as median (IQR) for continuous variables, or *n* (%) for categorical parameters.

Supplementary Table 3. Comparison of calorie and macronutrient intake between night and day shifts (*related to Figure 1*).

**Daily consumption (*n* = 66)**	**Reference values[1]**	**Day shifts**	**Night shifts**	**Difference**	**p-value**
Calories (kcal/day)	1861 to 2147 for women<br>2305 to 2672 for men	1939.7 ± 468.0	1751.3 ± 547.2	-163.8 ± 529.9	0.015
Carbohydrates (g/day)	n/a	208.8 ± 63.7	201.6 ± 65.3	-3.6 ± 59.2	0.62
Protein (g/day)<br>Relative to body weight (g/kg)	n/a<br>0.83 g/kg body weight	74.1 (56.9; 100.9)<br>1.26 (0.86; 1.58)	67.4 (53.0; 83.1)<br>1.05 (0.80; 1.36)	-9.6 ± 28.1<br>-0.15 ± 0.44	0.007<br>0.007
Fat (g/day)	n/a	86.0 ± 26.5	76.4 ± 31.9	-8.9 ± 29.3	0.017
Alcohol (g/day)	10 g for women<br>20 g for men	0.0 (0.0; 7.7)	0.0 (0.0; 0.0)	0.0 (-6.3; 0.0)	< 0.001
Fiber (g/day)	30	19.1 (15.6; 23.9)	17.8 (13.9; 21.1)	-1.93 ± 6.36	0.017
Carbohydrates (%)	45 to 60	43.3 ± 8.6	47.0 ± 9.0	2.6 (-1.3; 8.6)	< 0.001
Protein (%)	n/a	16.6 ± 4.0	15.8 ± 4.1	-0.6 ± 4.0	0.23
Fat (%)	20 to 35	39.7 ± 6.5	38.4 ± 7.5	-1.6 ± 7.5	0.09
Alcohol (%)	n/a	0.0 (0.0; 2.7)	0.0 (0.0; 0.0)	0.0 (-2.4; 0.0)	< 0.001
Time of last meal before 05:00h (h)	n/a	-7.70 (-8.47; -6.60)	-2.02 (-3.16; 1.00)	5.68 (4.10; 6.65)	< 0.001

Footnotes: Data are presented as mean ± SD or median (IQR), depending on the normal distribution of data. [1]Nutritional reference values for healthy adults aged between 18 and 65 in Switzerland[3].

Supplementary Table 4. Self-reported eating habits, alcohol and caffeine intake, and tobacco use between night and day shifts.

**Eating habits**, median (IQR)	***n***	**Day shifts**	**Night shifts**	**Difference**	**p-value**
Self-rated subjective appetite, mean ± SD	69	6.1 ± 2.0	5.5 ± 2.1	-0.7 ± 2.1	0.008
Changes in eating habits during night shifts, *n* (%)	72				
Longer eating duration			10 (13.9)		
Irregular meals			34 (47.2)		
Skipping meals			37 (51.4)		
Unhealthy food consumption			22 (30.6)		
Snacking			32 (44.4)		
Number of meals in the past 24 hours	70	3 (2; 3)	2 (2; 3)	0 (-1; 0)	0.01
Main meal of the day, *n* (%)	69				
Breakfast		6 (8.7)	3 (4.3)		
Lunch		31 (44.9)	7 (10.1)		0.004
Dinner		30 (43.5)	56 (81.2)		
Other		2 (2.9)	3 (4.4)		
Frequency of eating outside the usual mealtime in the past 24 hours	70	1.0 (0.5; 2.0)	2.0 (1.0; 3.0)	0.0 (-0.3; 1.0)	0.03
Frequency of eating in front of a screen in the past 24 hours	69	1.0 (0.2; 1.0)	1.0 (1.0; 2.0)	0.0 (0.0; 1.5)	0.001
Alcohol intake (units)	69	0 (0; 2)	0 (0; 0)	0 (-1; 0)	< 0.001
Caffeine intake (cups)	56				
Coffee		6 (2.75; 8)	5 (2; 8)	0 (-1; 1)	0.62
Tea		0 (0; 3)	1 (0; 2)	0 (-0.5; 1)	0.26
Caffeinated soda/ shake drinks		0 (0; 0)	0 (0; 0)	0 (0; 0)	0.39
Tobacco use throughout the shift period (numbers)					
Cigarettes	11	15 (4; 40)	6 (2; 30)	0 (-9; 4)	0.79
E-cigarettes	7	0 (0; 0)	0 (0; 0)	0 (0; 0)	0.91
Vaporizers	8	0 (0; 0)	0 (0; 0)	0 (0; 0)	0.93

Footnotes: Data are presented as mean ± SD or median (IQR), depending on the normal distribution of data.

Supplementary Table 5. Comparison of CGM metrics, actigraphy measurements and fasting insulin between night and day shifts (*related to Figures 2-3-5*).

	n	Day shifts	Night shifts	Difference	p-value
**CGM metrics**					
Mean glucose (mmol/L)	58	4.93 ± 0.67	4.78 ± 0.59	-0.19 ± 0.61	0.024
Coefficient of variation (%)	58	13.85 (11.83; 17.25)	15.47 (12.72; 18.96)	1.45 ± 4.52	0.018
Mean amplitude of glycemic variation (mmol/L)	58	1.31 (0.99; 1.65)	1.45 (1.12; 1.99)	0.21 ± 0.64	0.016
Baseline of baseline glucose cosinor model (mmol/L)	55	4.37 ± 0.70	4.11 ± 0.59	-0.29 ± 0.69	0.003
Amplitude of baseline glucose cosinor model (mmol/L)	55	0.53 (0.41; 0.68)	0.62 (0.46; 0.82)	0.08 ± 0.36	0.11
Damping coefficient	55	-0.60 (-1.02; -0.29)	-0.74 (-1.32; -0.29)	-0.31 (-0.73; 0.35)	0.10
Half-life (hours)	55	1.25 (1.01; 1.55)	1.23 (1.04; 1.51)	-0.06 (-0.20; 0.25)	0.88
Mean of meal heights (mmol/L)	55	0.90 (0.70; 1.16)	1.06 (0.86; 1.37)	0.20 (0.01; 0.42)	< 0.001
**Morning lab results**[1]					
Fasting insulin (mIU/L)	11	6.4 (5.2; 8.0)	6.1 (5.2; 7.5)	-0.7 (-1.9; 1.8)	0.86
HOMA-IR	11	1.40 (1.13; 1.72)	1.27 (1.02; 1.93)	-0.14 (-0.51; 0.54)	0.79
**Actigraphy measurements**					
Activity count	43	3.43 (2.94; 4.12)	3.11 (2.72; 4.00)	-0.21 (-0.66; 0.24)	0.12
Heart rate (beats/min)	43	77.54 (72.01; 82.99)	75.66 (67.39; 79.18)	-1.77 ± 4.63	0.018
Heart rate variability [log(RMSSD)]	43	3.58 ± 0.45	3.67 ± 0.42	0.07 (-0.10 to 0.23)	0.073

Footnotes: Data are presented as mean ± SD or median (IQR), depending on the data normal distribution. [1]Only those who had fasting measurements after the series of day and night shifts are shown. 66 participants were fasted for day shifts with median fasting insulin of 6.6 (IQR 5.3; 8.6) mIU/L, while only 11 participants were fasted at the end of their nights, explaining why the comparison is limited to a small subsample.

Abbreviations: CGM, Continuous Glucose Monitor; HOMA-IR, Homeostatic Model Assessment for Insulin Resistance, RMSSD, Root Mean Square of Successive Differences.

Supplementary Table 6. Comparison of sleep and fatigue between night and day shifts (*related to Table 2*).

**Sleep habits, sleep disturbances and fatigue (*n* = 72)**	**Day shifts**	**Night shifts**	**p-value**
Preferred sleep duration daily irrespective of shift work, hours	7.59 (1.07)		
Time of sleep onset	22:53h (22:00h; 23:00h)	09:00h (08:30h; 09:23h)	< 0.001
Time of wake-up	06:00h (05:30h; 06:30h)	14:30h (13:30h; 16:00h)	< 0.001
No. naps taken during shifts	0 (0; 0)	1 (0; 1)	< 0.001
Self-reported sleep quality, *n* (%)			< 0.001
Extremely badly	0	4 (5.6)	
Quite badly	2 (2.8)	16 (22.2)	
Moderately bad	20 (27.8)	29 (40.3)	
Quite well	35 (48.6)	20 (27.80	
Extremely well	15 (20.8)	3 (4.2)	
Perception of sleep sufficiency, *n* (%)			0.001
Nowhere near enough	7 (9.7)	14 (19.4)	
Could do with a lot more	9 (12.5)	26 (36.1)	
Could do with a bit more	41 (56.9)	21 (29.2)	
Get the right amount	14 (19.4)	11 (15.3)	
Get plenty	1 (1.4)	0	
Perceived restfulness after sleep, *n* (%)			< 0.001
Definitely no rested	4 (5.6)	12 (16.7)	
Not very rested	16 (22.2)	33 (45.8)	
Moderately rested	29 (40.3)	19 (26.4)	
Quite rested	20 (27.8)	8 (11.1)	
Extremely rested	3 (4.2)	0	
Difficulty falling asleep, *n* (%)			0.59
Never	24 (33.3)	21 (29.2)	
Rarely	16 (22.2)	26 (36.1)	
Sometimes	23 (31.9)	16 (22.2)	
Frequently	8 (11.1)	8 (11.1)	
Always	1 (1.4)	1 (1.4)	

**Sleep habits, sleep disturbances and fatigue (*n* = 72)**	**Day shifts**	**Night shifts**	**p-value**
Waking earlier than intended, *n* (%)			< 0.001
Never	28 (38.9)	7 (9.7)	
Rarely	21 (29.2)	7 (9.7)	
Sometimes	16 (22.2)	19 (26.4)	
Frequently	6 (8.3)	24 (33.3)	
Always	1 (1.4)	15 (20.8)	
Use of sleeping pills, *n* (%)			0.46
Never	68 (94.4)	66 (91.7)	
Rarely	2 (2.8)	1 (1.4)	
Sometimes	2 (2.8)	5 (6.9)	
Frequently	0	0	
Always	0	0	
Melatonin usage to aid sleep, *n* (%)			0.66
Never	61 (84.7)	63 (87.5)	
Rarely	2 (2.8)	4 (5.6)	
Sometimes	4 (5.6)	5 (6.9)	
Frequently	3 (4.2)	0	
Always	2 (2.8)	0	
Tiredness during shift, *n* (%)			< 0.001
Never	0	0	
Rarely	10 (13.9)	0	
Sometimes	32 (44.4)	15 (20.8)	
Frequently	26 (36.1)	30 (41.7)	
Always	4 (5.6)	27 (37.5)	

Footnotes: Questions adapted from a subset of questions in the Standard Shiftwork Index[2]. Data are presented as median (IQR) for continuous variables, or *n* (%) for categorical parameters.

Supplementary Figure 1.

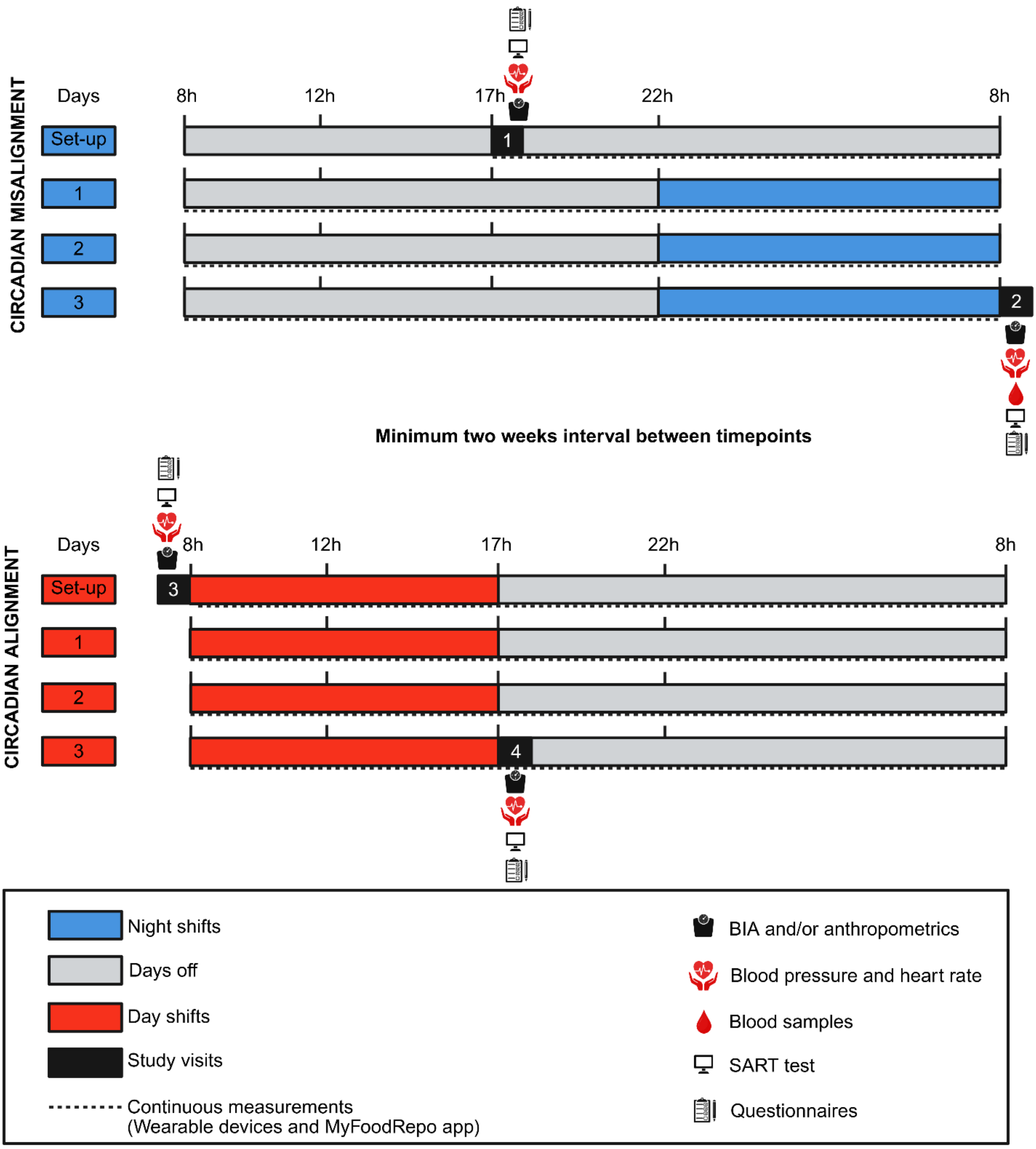

Supplementary Figure 1. Study design of the OPTI-SHIFT observational study.

The actual clock times of the shift start and end times could vary slightly according to the individual shift rotations. Abbreviations: BIA, Bioelectrical Impedance; SART, Sustained Attention to Response Task[4]. Created in BioRender. Jassil, F. (2026) https://BioRender.com/jrt4mfe

Supplementary Figure 2.

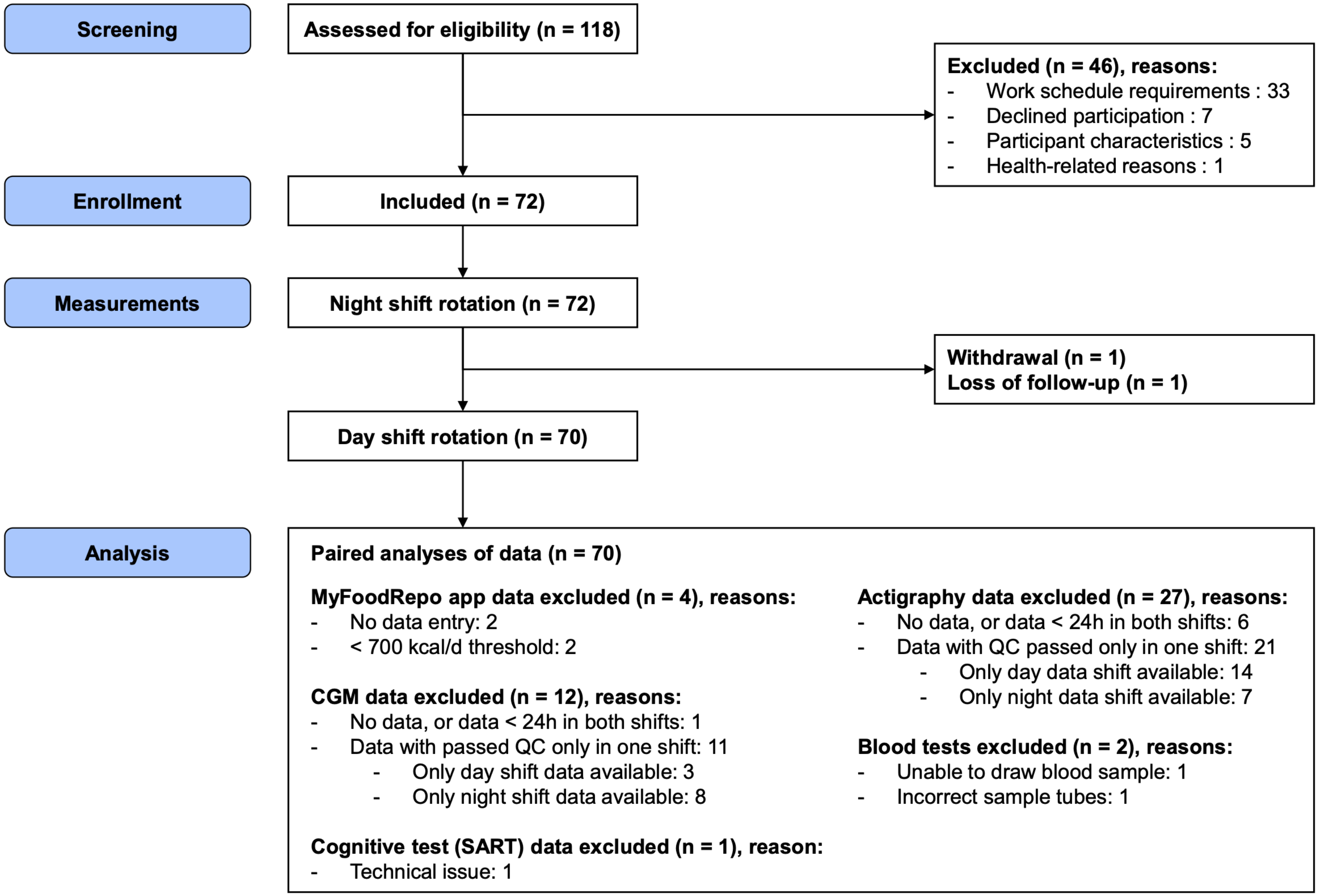

Supplementary Figure 2. Flow diagram of study participants from screening, assessment of inclusion/exclusion criteria, enrollment, measurements, to data analysis.

One participant started with day shift rotation first, followed by night shift rotation, for organisational reasons. The number of excluded individuals and the reasons for exclusion are detailed in the top-right textbox. The number of data excluded from analysis and the reasons are detailed in the bottom textbox. Abbreviations: CGM, Continuous Glucose Monitor; QC, Quality Control; SART, Sustained Attention to Response Task[4]

Supplementary Figure 3.

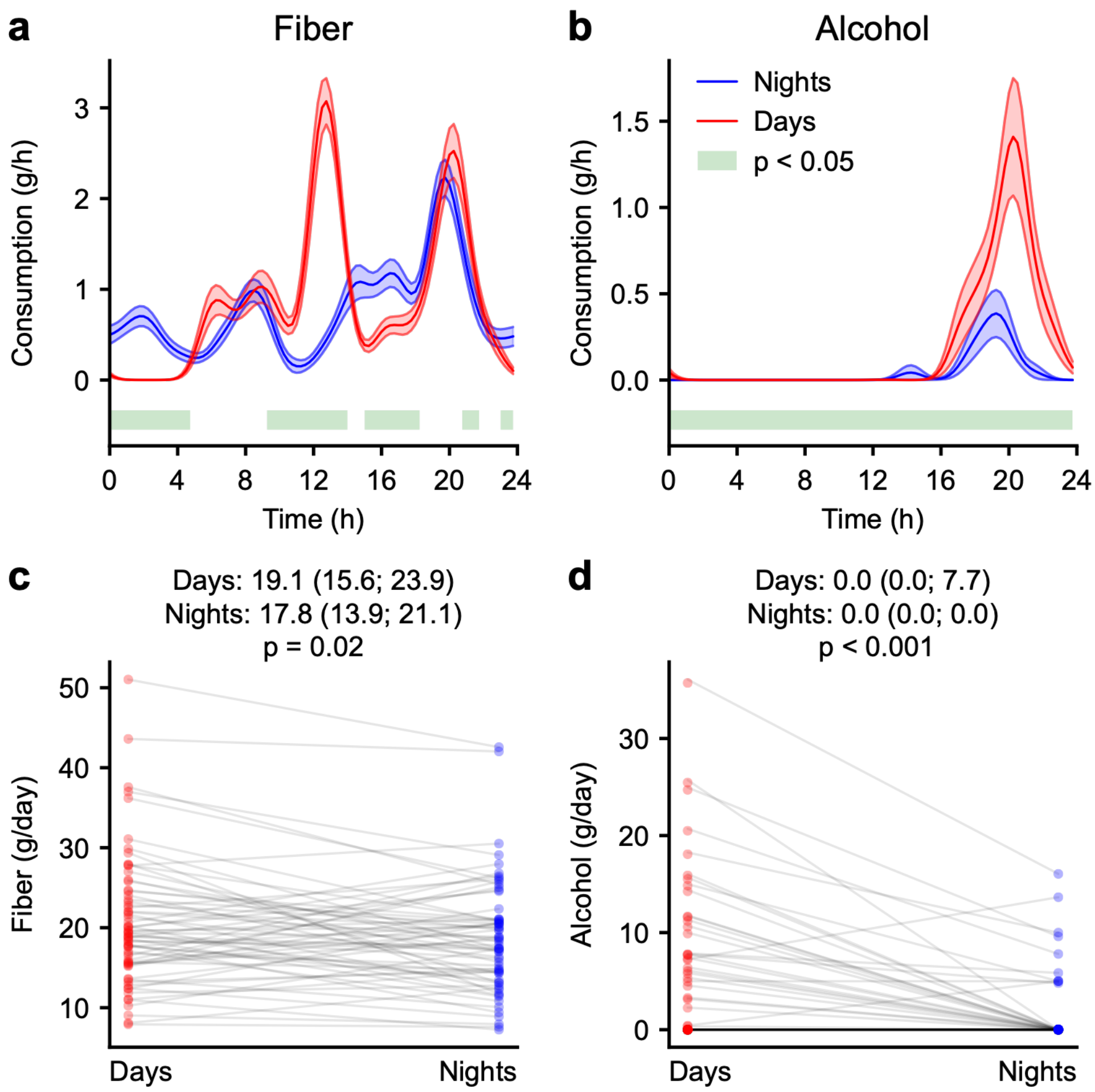

Supplementary Figure 3. Temporal distribution of fiber and alcohol consumption during day and night shifts (*related to Figure 1*).

(a-b) The mean consumption rate per hour for night shifts (blue) and day shifts (red) for fiber and alcohol, respectively. The shaded area represents the mean ± SEM, and the green bar represents significant group differences as assessed from $p < 0.05$ in a Wilcoxon non-parametric test, adjusted for multiple testing with the Benjamini-Hochberg procedure. (c-d) Individual average daily intake for the day and night shift period for fiber and alcohol, respectively. Group medians and IQR are indicated above each panel. *n* = 66 for all analyses.

Supplementary Figure 4.

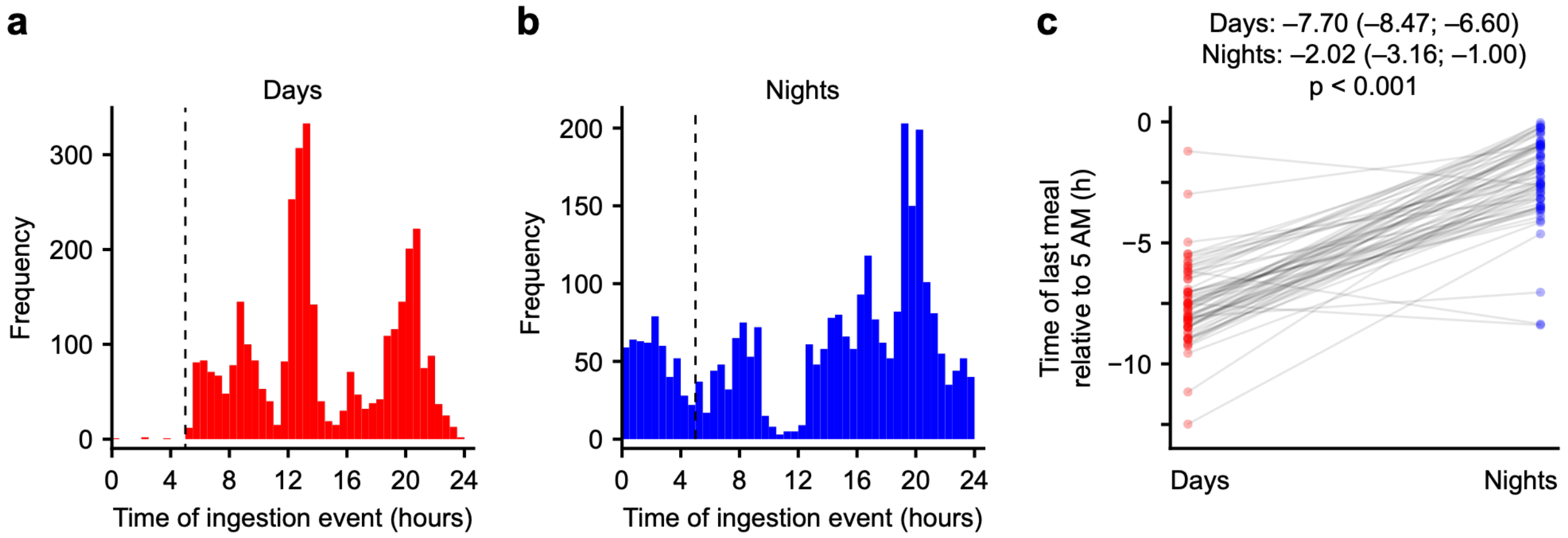

Supplementary Figure 4. Temporal distribution of ingestion events during day and night shifts.

(a-b) Temporal distribution of MyFoodRepo smartphone app food and drink entries (a) on day and (b) night shifts. The dashed line shows the 05:00h cutoff. (c) Individual changes in the time of the last calorie intake before 05:00h. On day shifts there is a trough in ingestion events between midnight and 05:00h, whereas this period still contains significant consumption during night shifts. On day shifts, the median time of the last calorie intake before 05:00h was –7.70 (IQR –8.47; –6.60) hours (i.e. a median time of 7.70 hours between the last calorie intake the previous day and 05:00h), while this time interval dropped to –2.02 (IQR –3.16; –1.00) hours for night shifts ($p < 0.001$).

Supplementary Figure 5.

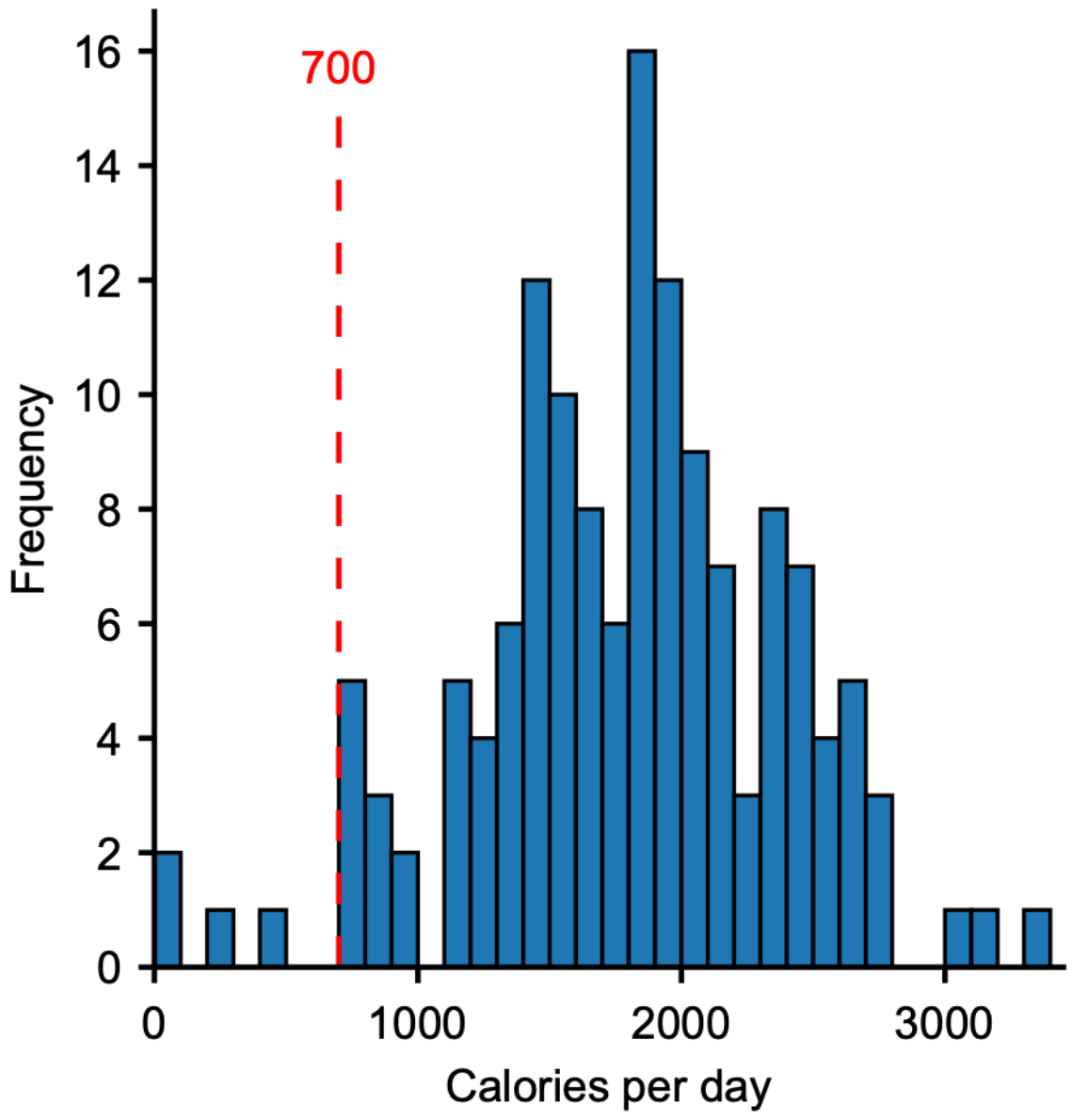

Supplementary Figure 5. Distribution of daily calorie intake recorded with the MyFoodRepo smartphone app over all shifts and participants of the study.

The dashed line shows the 700 kcal/day threshold used for filtering (see Methods).

References

1. Horne, J. A. & Östberg, O. A self-assessment questionnaire to determine morningness-eveningness in human circadian rhythms. *Int. J. Chronobiol.* **4**, 97–110 (1976).
2. Barton, J., Spelten, E., Totterdell, P., Smith, L. & Folkard, S. Is there an optimum number of night shifts? Relationship between sleep, health and well-being. *Work Stress* **9**, 109–123 (1995).
3. Federal Food Safety and Veterinary Office (FSVO). Recommendations for healthy adults aged between 18 and 65. https://valeursnutritives.ch/en/nutrients/ (2025).
4. Robertson, I. H., Manly, T., Andrade, J., Baddeley, B. T. & Yiend, J. 'Oops!': performance correlates of everyday attentional failures in traumatic brain injured and normal subjects. *Neuropsychologia* **35**, 747–758 (1997).
